# Extended Restitution Between Sessions Does Not Enhance the Benefits of 12 Weeks Exercise‐Based Treatment for Patellar Tendinopathy: A Randomized Controlled Clinical Trial (The TEREX Trial)

**DOI:** 10.1111/sms.70235

**Published:** 2026-03-08

**Authors:** Anne‐Sofie Agergaard, Rene B. Svensson, Rikke Hoeffner, Syed Zahra Gillani, S. Peter Magnusson

**Affiliations:** ^1^ Institute of Sports Medicine Copenhagen, Department of Orthopedic Surgery Copenhagen University Hospital ‐ Bispebjerg and Frederiksberg Copenhagen Denmark; ^2^ Center for Healthy Aging, Department of Clinical Medicine University of Copenhagen Copenhagen Denmark; ^3^ Department of Physical and Occupational Therapy Bispebjerg and Frederiksberg University Hospital Copenhagen Denmark; ^4^ Center for Fast Ultrasound Imaging, Department of Health Technology Technical University of Denmark Lyngby Denmark

**Keywords:** loading‐based treatment, patella tendon, restitution time from loading, tendinopathy

## Abstract

Loading intervention is the predominant treatment strategy for tendinopathy and the response may depend on restitution time between loading exposure. Therefore, the purpose of this study was to investigate if a 12‐week exercise‐based rehabilitation regime for patellar tendinopathy is influenced by restitution time. We hypothesized that longer restitution would yield greater improvements in clinical outcomes, tissue structure, and function compared to shorter restitution. Fifty‐two participants with chronic patellar tendinopathy were randomized to a short restitution group (SR, 3 exercise days/week) or an extended restitution group (ER, 1 exercise day/week). For both groups, each session consisted of resistance exercises (Leg press and knee extension) with a load starting at ~60% of 1 RM and progressing to ~75% of 1 RM and impact activities (running and jumping), restricted in both groups. Function and symptoms (VISA‐P), tendon pain during activity (NRS), tendon function (functional tests), and ultrasound (tendon vascularization and swelling) were assessed before and after the intervention. Self‐reported improvement and satisfaction with function and treatment were measured at week 12. The results revealed that ER was not superior to SR for any of the outcomes. Both groups attained significant improvements in clinical outcomes and muscular strength. Conversely, there were no improvements in jumping height or tendon structure. Moreover, there were no group differences in self‐reported improvement or satisfaction with function or treatment. In conclusion, there was no superior effect of 1 exercise day/week compared to 3 exercise days/week. Both groups demonstrated equal clinical and muscular strength improvement after 12 weeks without any improvement in structure or jumping performance. No group differences were found in self‐reported improvement or satisfaction with function or treatment at week 12.

## 1 Introduction

Patellar tendinopathy is a common overuse injury, with a prevalence from 9% in non‐elite to 14% in elite athletes, and up to 45% among athletes performing explosive sports [1, 2]. Notably, this type of injury is a substantial clinical challenge because it often impacts function and work ability long‐term (months to years) [3, 4], and is consequently a sizeable economic burden on society [5]. Loading‐based treatment is currently the preferred treatment for tendinopathy [6], although the optimal loading configuration remains largely unknown [7].

The beneficial outcomes of a stretch‐shortening exercise program first attracted attention as a treatment option of tendinopathy back in the 80´s [8], and since then varying treatment regimens for tendinopathy, including different loading configurations, have been suggested. In the 90's eccentric exercise [9] gained considerable popularity despite a lack of robust clinical or mechanistic evidence favoring this specific modality [10, 11]. More recently new loading‐based exercise regimes, such as heavy slow resistance training, have emerged. Evidence from clinical trials [12, 13] and from basic science [14, 15] have suggested that load magnitude is an important component in attempts to optimize the treatment response. However, high load magnitude does not appear to be superior to moderate load magnitude with respect to clinical outcome, tendon structure, and the function of tendinopathic patellar tendon [16]. Importantly, both loading regimes resulted in clinical improvements in the short and long term, although complete recovery was not achieved. However, the impact of other exercising variables, such as restitution time, on the outcome and lack of full recovery remains to be determined.

The importance of restitution for muscle tissue between training sessions is a well‐accepted concept. For example, resistance training to achieve increased strength can at times be counterproductive if performed > 2 times per week [17]. However, such considerations have yet to be incorporated in the rehabilitation of tendinopathies. It has previously been shown that tendon cells are metabolically active in response to loading [18], but it is unknown whether rest is required for the purpose of restoring energy deposits considering the relatively low metabolism. On the other hand, the anabolic response to loading is sustained in tendons up to several days following an exercise bout [19], which could indicate the need for a post exercise resting period [20]. Most eccentric exercise protocols for tendinopathy management are performed every day without rest periods [9, 21], however, the net balance between synthesis and degradation of collagen in response to a single bout of loading has been suggested to be negative up to 36 h after exercising with positive synthesis lasting from 36 to 72 h [20], which prompts the question if increased restitution from loading will yield a more positive clinical outcome, structure and function of tendon in patients with patellar tendinopathy.

Therefore, the purpose of the present study was to investigate if the restitution time in a 12‐week exercise‐based rehabilitation regime for patellar tendinopathy influences the outcome. We hypothesized that a longer restitution from loading (1 exercise day per week) would yield a greater positive clinical outcome and tissue structure and function in patients with patellar tendinopathy compared to shorter restitution (3 exercise days per week) when impact activities were restricted in both groups.

## 2 Materials and Methods

### 2.1 Trial Design

The TEREX study, conducted at Bispebjerg and Frederiksberg Hospital, Copenhagen, Denmark, has two phases; the first phase was a prospective randomized controlled, single‐blinded, superiority trial reported in the present paper. The second phase includes an observational follow‐up from 12 weeks to the study endpoint at 52 weeks. Outcomes from the observational study will be published separately. Ethical approval was obtained from the regional Ethics Committees for medical research (H‐22031880) and all participants provided written informed consent. The study was pre‐registered on ClinicalTrials.gov (NCT05731037) before inclusion of the first participant. This report follows the Consolidated Standards of Reporting Trials (CONSORT) guidelines and the Template for Intervention Description and Replication (TIDieR) checklist for intervention description [22].

### 2.2 Patient Involvement

Patient partners were involved in the planning phase of this study to capture the patient perspective on the idea and purpose of the study, whether the intervention was feasible and the time requirement of participating in the study. The patient partners participated voluntarily in the process and the collaboration between patients and professionals in the project follows the European League Against Rheumatism recommendations (EULAR) [23] for the inclusion of patients' representatives in scientific projects.

### 2.3 Participants

Participants were recruited through the Sports Clinic at Bispebjerg and Frederiksberg Hospital and via advertisements on social media and the internet. Inclusion criteria were sports‐active men and women, age 18–60 years, BMI 18.5–30, patellar tendon pain duration of 3–24 months, and a clinical diagnosis of unilateral or bilateral patellar tendinopathy by experienced sports physicians based on defined clinical findings (symptoms localized to the patellar tendon region on loading and pain or discomfort of the inferior pole of the patella at palpation). Further, the clinical diagnosis required at least one of the following three changes to be present on ultrasonography: thickening of the anterior–posterior diameter of the symptomatic area compared with the mid tendon level, presence of Power Doppler signal in the tendon on the symptomatic side, and a hypoechogenic area corresponding to the symptomatic area of the tendon. If the participant had bilateral symptoms, the most symptomatic tendon selected by the participant was defined as the study tendon. If both tendons were equally symptomatic, the tendon with the most severe thickening of the tendon compared with the mid‐tendon level was defined as the study tendon. Exclusion criteria were previous surgery in the knee on the ipsilateral side, corticosteroid injection in the patellar tendon on the ipsilateral side within the last 6 months, any confounding diagnosis to the knee joint, known arthritis, known diabetes, smoking, inability to follow rehabilitation or complete follow‐ups, having enrolled in a resistance‐based rehabilitation program for the affected patellar tendon within the previous 3 months, and having a job where it was not feasible to avoid pain‐provoking tasks.

### 2.4 Randomization and Blinding

After collection of demographic information and all baseline assessments, participants were randomly allocated to either the short restitution group (SR) or the extended restitution group (ER). The SR group, with three resistance training sessions per week, constitutes the currently accepted rehabilitation program for patellar tendinopathy at the department and was therefore considered the control group. A randomization procedure was performed using a computer‐generated block randomization (blocks of 4 or 6) procedure. The allocation ratio was 1:1 and participants were stratified according to sex and symptom duration. Allocation concealment was ensured by restricting access to the randomization module in REDcap to two independent colleagues who were not involved in the follow‐up measurements.

The participants and the sports physiotherapists delivering the interventions could not be blinded to treatment allocation. Outcome assessor (AA) was blinded to treatment allocation, and participants were requested not to disclose their allocation when outcomes were assessed. Moreover, all patient‐reported outcomes were obtained electronically and blinded for members of the research team using REDCap.

### 2.5 Interventions

The exercise program was identical for both groups apart from the SR‐group performing the resistance exercises three times per week and the ER‐group only once per week (increased restitution time). For the SR‐group, there should be at least 48 h rest and for the ER‐group, 96 h between each training session.

The exercise program lasted for 12 weeks and was performed individually in a commercial fitness center. One session in weeks 1, 3, and 6 was carried out under individual supervision of a sports physiotherapist and delivered face‐to‐face at the physiotherapy department at Bispebjerg and Frederiksberg Hospital. In weeks 2 and 4, the participants were reminded of changing the training load by a text message, and in week 9, the physiotherapist followed up by phone on the training intervention, training compliance, and compliance with the impact‐load reduction regime.

The intervention program included resistance exercise therapy and load reduction from impact activities such as running and jumping.

#### 2.5.1 Load Reduction

Participants in both groups were not allowed to perform impact activities (running and jumping), outside of the prescribed treatment, that could provoke their patellar tendon symptoms. However, they were encouraged and guided by the supervising physiotherapist in how to perform non‐impact activities (e.g., biking, swimming, rowing, strength training not involving the quadriceps muscle and any part of their normal training not including impact on the patellar tendon).

#### 2.5.2 The exercise program

For both groups, each session consisted of one bilateral leg press exercise and one unilateral knee extension exercise (Supporting Information), with each lasting 6 s (3 s for the concentric and eccentric phases, respectively). The leg press was performed from 10° to 90° of knee flexion. Knee extension was performed from 100° to 30° of knee flexion. An increase in load was recommended when the participant was able to perform 2 repetitions more than the desired number within the limits of pain. Participants were instructed to warm up by 5 min of cycling on a stationary bike ergometer with moderate intensity (corresponding to 11–15 on Borg Rating of Perceived Exertion Scale; “somewhat hard”) before the exercise protocol.

The program was started at a load of 15 repetition maximum [RM] ~60% of 1 RM and progressed to 10 RM (~75% of 1 RM) during the first 3 weeks, which was maintained throughout the rest of the intervention. The volume started at 3 sets in each exercise, progressing to 4 sets after 6 weeks, with 1–2 min of rest between sets. Information about the loading protocol is summarized in Table 1.

**TABLE 1:** Loading protocol, identical for the two intervention groups.

Exercise protocol				
Week	1	2–3	4–5	6–12
Sets and reps	3 × 15	3 × 12	3 × 10	4 × 10
∼ % of 1 RM	60	70	75	75

Abbreviation: RM, repetition maximum.

The exact training load for each exercise was estimated based on the RM listed in Table 1. Each exercise was initiated with loading that achieved muscle fatigue within 15 repetitions in each set. Pain during these exercises (numeric rating scale [NRS]) was accepted to reach 5 on the 0–10 NRS but pain and discomfort should not increase following cessation of training and if any training‐induced pain did not subside 3–4 h after the session, the load was reduced during the next session. Participants in both groups were instructed to only perform other non‐impact activities if patellar tendon pain could be maintained below 3 on the 0–10 NRS. If pain intensity exceeded 3 on the NRS, the participant was recommended to stop the activity.

Compliance with exercise and activity modification was tracked using an electronic training diary. Participants were asked to record the number of sessions and load of the treatment exercises completed and whether they performed running, jumping, or other activities outside of the intervention. For detailed information of the intervention, following the (TIDieR) checklist for intervention description see Supporting Information.

### 2.6 Follow‐Up Evaluation

Outcome measurements were obtained 3 to 4 days before and after the 12‐week intervention period. The primary outcome was the change in Victorian Institute of Sports Assessment –patella [VISA‐P] score from baseline to 12 weeks. The examination order was identical for all evaluations, which always started with questionnaires, followed by ultrasound, and 5 min warm‐up on a bicycle ergometer before functional testing.

### 2.7 Patient‐Reported Outcome Measures

Participants' symptoms, function and ability to participate in sports were evaluated using the diagnose specific questionnaire VISA‐P. The VISA‐P consists of 8 questions, and a score of 100 points indicating the person is asymptomatic and without limitations and lower scores indicating more symptoms and limitations of function and activity [24]. The minimal clinically important difference for the VISA‐P in athletes with patellar tendinopathy is considered to be 13 points [25].

Given that VISA‐P has recently been shown to have psychometric issues, a truncated version (only including Item 2–6), which has been shown to have more adequate psychometric properties in a recent analysis, was also included [26]. In addition, maximal tendon pain during physical activity, daily activities and rest were evaluated on a 0 to 10 NRS, with 10 being the worst imaginable pain and 0 denoting no pain. Furthermore, a 5‐point Likert scale ranging from ‘very satisfied’ to ‘very unsatisfied’ was completed with the following 2 questions in relation to their tendinopathy: (1) How satisfied are you with your level of function in everyday activities if your current condition does not change and (2) How satisfied are you with your level of function in sports and physical activities if your current condition does not change? Patients were dichotomized as satisfied if they rated themselves as ‘very satisfied’, ‘satisfied’ or ‘neutral’ or as unsatisfied if they rated themselves ‘unsatisfied’ or ‘very unsatisfied’. Both VISA‐P, tendon pain evaluation and satisfaction with function were completed at baseline and at 12 weeks follow‐up. Weekly sport participation (hours) during the preceding week was evaluated before the injury (recall), at baseline and 12 week follow up. At 12 weeks, the Global Rating of change (GROC) was used to measure patient self‐reported improvement on a 7‐point Likert scale ranging from ‘much improved’ to ‘much worse’. Patients were dichotomized as improved if they rated themselves as ‘much improved’, ‘improved’ or ‘slightly improved’ (categories 5, 6 and 7) and categorized as not improved if they rated themselves from ‘unchanged’ to ‘much worse’ (categories 1 to 4).

### 2.8 Functional Evaluation

Isokinetic knee‐extension torque was measured with the participants seated with the hip flexed to 85° and the knee flexed at 90° (Biodex Multi‐joint System 4 Pro; Biodex Medical Systems). Warm‐up consisted of 1 set of 4 dynamic knee extensions to get accustomed to the speed and range of motion, followed by three repetitions with approximately 50%, 80%, and 100% of maximal effort, respectively. The participants performed a series of 3 sets with 4 repetitions of maximal dynamic knee extensions from 100° to 20° of knee flexion at a speed of 60° per second. There was a rest period of 60 s between each set, and the participants reported pain using the NRS upon completion of each test set. Both legs were tested, starting with the one selected as study tendon at inclusion. The outcome of the test was the maximal peak torque across all test sets normalized to body weight.

The single‐leg decline squat (SLDS) [27], which is a reliable patellar tendon pain provocation test, was used to examine pain during function. Participants performed a squat on a 25° decline board and reported pain using the NRS upon completion. The participants were instructed to stand on one leg with their hands placed at the waist and to keep the trunk vertical and heels in contact with the board. The SLDS was performed until 50° of knee flexion, and participants returned to the starting position at a self‐determined speed as described previously [28]. One practice trial was performed before the two tests, with a 1‐min rest period between the tests. The NRS score was collected immediately after each trial, and the mean of the two scores was used for analysis.

Counter‐movement jump (CMJ) was used to examine jump function at baseline and 12 weeks. CMJ tests were performed on two portable force plates (Vald Performance, Brisbane, Queensland, Australia), and the vertical jump height was estimated from the impulse. The CMJ was started on straight legs and with hands akimbo during the entire movement. The test was performed both on two and one leg. The participants performed one practice trial followed by three maximal jumps and the NRS score was collected immediately after each trial. The max jump height of the three jumps was used for analysis.

### 2.9 Ultrasonography

All participants were instructed to refrain from strenuous physical activity 24 h before their ultrasound examination. The examination was performed at baseline and 12 weeks follow‐up by the same experienced assessor (AA) using a General Electric LOGIQ E10 ultrasound machine (General Electric, Wauwatosa, WI, USA) and an ML6‐15 transducer (50 mm length). The same software settings were used for all examinations, and all imaging was performed twice with the probe removed off the skin and relocated between recordings. One investigator conducted all analyses blinded to intervention allocation and side.

The power doppler flow (PD) in the patellar tendon was acquired using Microvascular Imaging (MVI) with the depth fixed at 3.3 cm, a color Doppler frequency of 8 MHz, and a pulse repetition frequency of 183 Hz. The examination was performed with the participants lying supine with a completely extended and relaxed knee. The investigator applied minimum transducer pressure during scanning. The transducer was placed at a 90° angle and moved medially to laterally to locate the maximum Doppler signal. At this location, two 3‐s sine loops were recorded in the sagittal plane.

The anterior–posterior patellar tendon thickness was measured with B‐mode ultrasonography (depth 3.3 cm, a frequency of 15 MHz, and a gain of 56). The examination was performed with the participant in a seated position with 90° of hip and knee flexion. The transducer was placed at a 90° angle (not rotated along the tendon surface) and moved medially to laterally to find the place where the tendon was thickest, and the two images were recorded.

Fiji/ImageJ (Version 2.14.0/1.54f; National Institutes of Health) was used for quantitative analysis. A custom macro was used to analyze the frame containing the largest area of Doppler activity in each series. Doppler was only included if it was localized within the tendon. The image with the largest Doppler area was used for further analysis. The AP patellar tendon thickness was measured 0.5 cm distal from the patellar apex. The specific measuring site and method were followed as previously described [16]. The maximum of the AP thickness from the two recorded images was used for further statistical analysis.

### 2.10 Sample Size

The study was powered based on previous data. A within‐subject standard deviation of the primary outcome (VISA‐P) of 12.8 after 12 weeks was expected [16]. A sample size analysis revealed that each group should contain *n* = 18 to detect a 13 points difference [25] (minimal clinically important difference) on VISA‐P score with an alpha level of 0.05 and a power/beta level of 0.80. To account for a 20% dropout rate and an estimated compliance rate of 75% (percentage of participants completing > 80% of intervention) based on previous data, a total of 26 participants were recruited to each group to ensure sufficient numbers for both intention‐to‐treat and per‐protocol analysis.

### 2.11 Statistical Analysis

All data were analyzed using GraphPad Prism (Version 10.4.0 for Mac; GraphPad Software) following a predefined statistical analysis plan registered on ClinicalTrials.gov (NCT05731037). Results are reported as the mean ± SEM unless otherwise noted. Baseline demographic data and participant compliance were analyzed by using the unpaired Student *t*‐test. Outcome parameters were analyzed by a mixed‐linear model with two independent variables: Time (baseline and 12 weeks) and group (ER and SR), including interactions. Satisfaction and GROC data were described by number and %. For outcomes only analyzed at 12 weeks (treatment satisfaction and GROC), dropouts were given a value of zero in the calculation of % participants satisfied or improved. Further, Chi‐square (and Fisher's exact) test was performed for all analysis based on a contingency table. Correlations between structural and clinical outcomes were analyzed using Pearson's correlations for parametric data and Spearmann's rho for non‐parametric. The significance level for all tests was set to *p* < 0.05. All analyses were performed as intention‐to‐treat. Results of a compliance based (performed at least 80% of the prescribed exercise sessions and have complied with the load‐reduction) per protocol subgroup are available in Supporting Information.

## 3 Results

### 3.1 Participants

**FIGURE 1:**
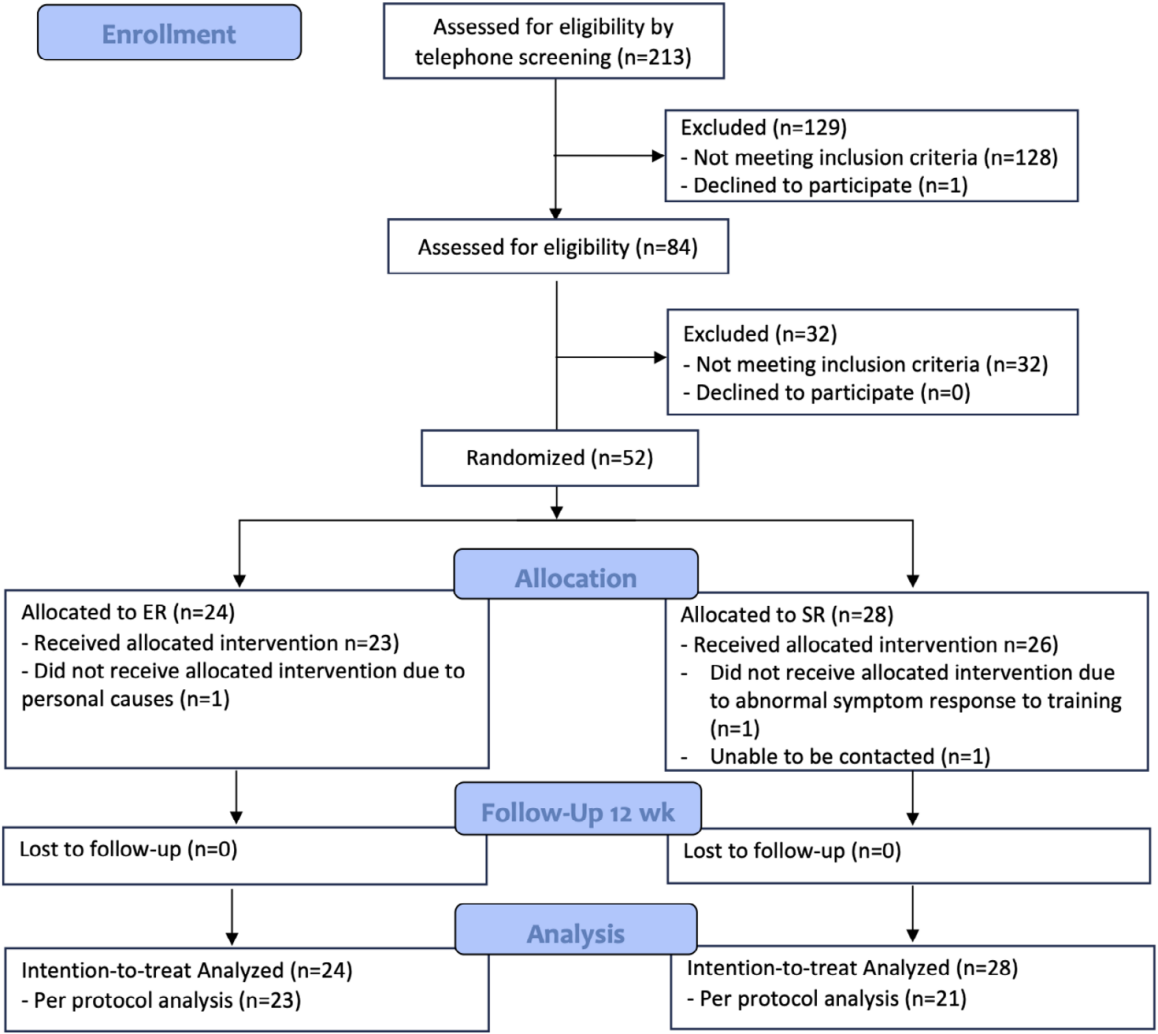
CONSORT (Consolidated Standards of Reporting Trials) flowchart for the primary outcome of the Victorian Institute of Sport Assessment–Patella (VISA‐P). SR, Short restitution group; ER, Extended restitution group.

From March 2023 through July 2024, 213 individuals were screened for eligibility (Figure 1), 161 were ineligible for inclusion. Thus, 52 participants were randomized into 1 of 2 groups. During the intervention, there was one dropout in each of the intervention groups (personal causes and unable to be contacted), and one participant in the SR group was excluded due to abnormal symptom (significant doppler and thickening throughout the entire length of the tendon) in response to training. Baseline characteristics were similar in the two groups, except for a significantly higher BMI in the ER group (Table 2). All participants were recreational athletes, with a large number (*n* = 14) involved in soccer and other preferred sports: running including orienteering (*n* = 11), strength training including CrossFit (*n* = 9), volleyball (*n* = 4), tennis (*n* = 2), handball (*n* = 2), badminton (*n* = 2), martial arts (*n* = 2), squash (*n* = 1), paddle (*n* = 1), basketball (*n* = 1), skateboarding (*n* = 1), cheerleading (*n* = 1), and dodgeball (*n* = 1).

**TABLE 2:** Baselines characteristics.

Variable	SR (*n* = 28)	ER (*n* = 24)
Age, y	30.6 ± 8.0 (21–56)	33.2 ± 9.1 (22–54)
Male/female, *n*	21/7	20/4
Height, cm	183.3 ± 9.8	181.9 ± 8.0
Weight, kg	80.3 ± 13.4	87.9 ± 16.1
Body mass index (kg/m^2^)	23.9 ± 2.2	26.4 ± 3.4
Symptom duration, mo	12.0 ± 7.0 (3–24)	11.6 ± 7.0 (4–24)
Sport participation, h/wk	8.8 ± 4.8 (2–20)	8.3 ± 3.5 (2–15)
Unilateral/bilateral injury, *n*	19/9	10/14

*Note:* Values are expressed as mean ± SD (range) unless otherwise noted.

Abbreviations: ER, Extended restitution group; SR, Short restitution group.

### 3.2 Compliance

The mean training session compliance rate was significantly different between the two groups (*p* = 0.04) with a compliance rate of 81% ± 4% for the SR group and 93% ± 4% for the ER group. The supervised session compliance rate was 90% ± 4% for the SR and 90% ± 5% for the ER for attendance sessions (week 1, 2, and 3) (*p* = 0.98) and 92% ± 7% for the SR and 96% ± 4% for the ER for phone supervision (week 9) (*p* = 0.13). The number of participants who did not comply with the load‐reduction (avoiding intense running or jumping outside of the intervention protocol) for at least 10 of the 12 weeks was two in the SR group and none in the ER group.

All data from the per‐protocol analyses (participants who fulfilled at least 80% of the prescribed training sessions and have complied with the load‐reduction) are shown in the Supporting Information (Tables S1–S6 and Figure S1) and were similar to those of the intention‐to‐treat analyses.

### 3.3 Clinical

**FIGURE 2:**
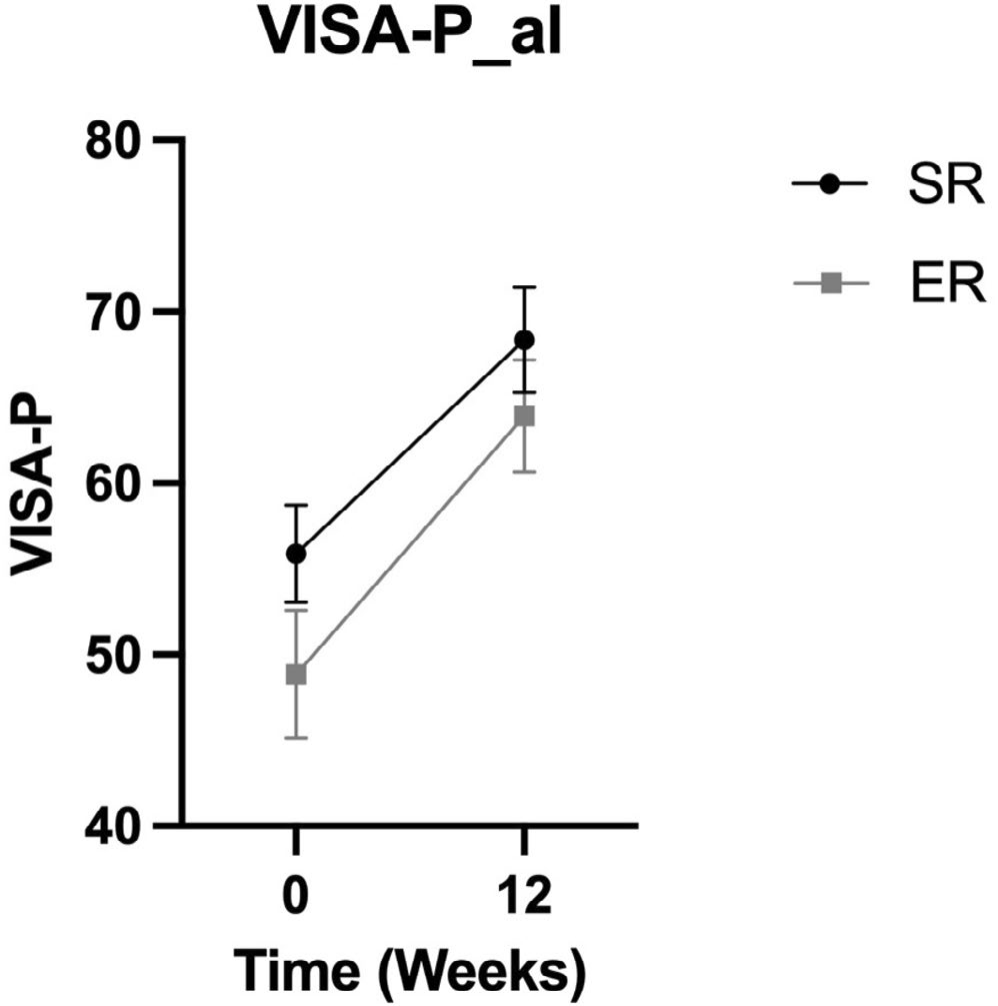
Clinical improvement in VISA‐P. Values are presented as mean ± SEM at baseline (0wk) and after intervention (12wk) for the two intervention groups. VISA‐P, Victorian Institute of Sports Assessment–Patella, SR, Short restitution group; ER, Extended restitution group. A mixed effect model was performed for all analysis with time and group as main factors. Alpha level set at *p* < 0.05. *P*‐values group (0.16), time (< 0.0001), and interaction (0.54).

There was no significant group (*p* = 0.16) or interaction (*p* = 0.54) effect, but a significant effect of time (*p* < 0.0001) with increased VISA‐P score after 12 weeks (Table 3 and Figure 2). Similarly, for truncated VISA‐P, there was no group or interaction effect, but a main effect of time.

**TABLE 3:** Clinical Results.

	SR (*n* = 28)	ER (*n* = 24)	*P*		
			Group	Time	Group × Time
VISA‐P, Point					
0 weeks	55.9 ± 2.8 (50.1 to 61.7)	48.8 ± 3.7 (41.1 to 56.6)	0.16	< 0.0001	0.54
12 weeks	68.3 ± 3.1 (62.0 to 74.7)	63.9 ± 3.3 (57.2 to 70.7)			
VISA‐P truncated, Point					
0 weeks	32.5 ± 1.9 (28.7 to 36.3)	28.3 ± 2.2 (23.9 to 32.7)	0.07	< 0.0001	0.91
12 weeks	42.6 ± 1.2 (42.6 to 40.1)	39.0 ± 1.6 (35.7 to 42.4)			
SLDS, NRS					
0 weeks	5.1 ± 0.6 (4.0 to 6.3)	6.5 ± 0.5 (5.4 to 7.5)	0.05	< 0.0001	0.68
12 weeks	2.7 ± 0.5 (1.5 to 3.8)	4.1 ± 0.6 (2.9 to 5.3)			
Pain, NRS					
Physical activity					
0 weeks	5.7 ± 0.5 (4.7 to 6.6)	6.3 ± 0.5 (5.3 to 7.3)	0.24	< 0.0001	0.85
12 weeks	2.6 ± 0.3 (2.1 to 3.2)	3.3 ± 0.4 (2.4 to 4.1)			
Daily activity					
0 weeks	3.9 ± 0.4 (3.1 to4.7)	4.6 ± 0.5 (3.6 to 5.6)	0.13	< 0.0001	0.69
12 weeks	1.6 ± 0.3 (1.1 to 2.1)	2.6 ± 0.5 (1.5 to 3.6)			
Rest					
0 weeks	2.8 ± 0.5 (1.9 to 3.7)	3.4 ± 0.5 (2.3 to 4.4)	0.17	< 0.0001	0.74
12 weeks	1.0 ± 0.2 (0.5 to 1.4)	1.8 ± 0.4 (1.0 to 2.6)			
CMJ (bilat)					
0 weeks	2.4 ± 0.4 (1.6, 3.2)	2.4 ± 0.7 (1.0, 3.8)	0.85	< 0.0001	0.74
12 weeks	0.7 ± 0.2 (0.3, 1.1)	0.8 ± 0.3 (0.2, 1.4)			
CMJ (test Leg)					
0 weeks	3.0 ± 0.4 (2.2, 3.8)	4.3 ± 0.6 (3.1, 5.6)	0.02	< 0.0001	0.62
12 weeks	0.8 ± 0.2 (0.4, 1.2)	1.8 ± 0.4 (1.0, 2.6)			
Muscle strength tets (test leg)					
0 weeks	3.8 ± 0.5 (2.8, 4.8)	4.5 ± 0.6 (3.3, 5.8)	0.11	< 0.0001	0.46
12 weeks	1.7 ± 0.3 (2.4, 1.0)	2.8 ± 2.8 (1.9, 3.7)			

*Note:* Values are presented as least 55.9 mean ± SD (95% CI). Mixed effect model was performed for all analysis with time and group as main factors. Alpha level set at *P* < 0.05.

Abbreviations: CMJ, countermovement jump; ER, Extended restitution group; NRS, numeric rating scale; SLDS, single‐leg decline squat; SR, Short restitution group; VISA‐P, Victorian Institute of Sports Assessment‐ Patella.

For the NRS score for physical activity, daily activity, and rest there were significant reductions in pain with time (*p* < 0.0001) but no significant group or interaction effects (Table 3). Figures showing individual participants' data for VISA‐P, VISA‐P truncated, and pain during daily activity can be found in Supporting Information, (Figure S2).

There was a significant main effect of time (*p* < 0.0001) with reduced NRS score during SLDS test from baseline to 12 weeks. No significant interaction effect was detected (*p* = 0.68), but there was a main effect of group (*p* = 0.05) with greater NRS scores in the ER group (Table 3). The detected group effect disappears when adjusted for baseline values (*p* = 0.29).

For physical activity level, there were no significant group or interaction effects, but a significant effect of time (*p* < 0.0001) with decreasing sport participation (hr/wk) from before injury (recorded as recall at baseline) to 12 week (Table 4).

**TABLE 4:** Sports participation (hr/wk).

	SR (*n* = 28)	ER (*n* = 24)	*P*		
			Group	Time	Group × Time
Before injury	8.7 ± 0.7 (7.2, 10.2)	8.4 ± 0.9 (6.5, 10.3)	0.9	< 0.0001	0.9
0 weeks	5.7 ± 0.8 (4.1, 7.3)	6.0 ± 0.7 (7.5, 6.0)			
12 weeks	3.8 ± 0.7 (2.4, 5.2)	4.2 ± 0.6 (5.4, 4.2)			
Δ before injury to 0 week	3.0 ± 0.8 (1.0, 5.0)	2.4 ± 0.9 (0.3, 4.6)			
Δ before injury to 12 weeks	4.8 ± 0.8 (2.8, 6.8)	4.2 ± 0.9 (2.1, 6.4)			

*Note:* Values are presented as least mean ± SEM (95% CI). Mixed effect model was performed for all analysis with time and group as main factors. Alpha level set at *p* < 0.05.

Abbreviations: ER, Extended restitution group; SR, Short restitution group; Δ, change in time interval.

Regarding satisfaction with function, there was no group effect for daily activity or sport and physical activity respectively. Likewise, no time effect could be detected in any of the two groups for daily activity (SE group, *p* = 0.18; ER, *p* = 0.15) or for sport and physical activity (SE group, *p* = 0.05; ER, *p* = 0.11) (Table 5). For satisfaction with treatment (only measured at 12 weeks) there was no group difference (*p* < 0.9999). Similarly, in regard to improvement following treatment, measured on a GROC scale at 12 weeks follow‐up, there was no group effect (*p* = 0.23).

**TABLE 5:** Satisfaction and Improvement.

	SR (*n* = 28)	ER (*n* = 24)	*P* (group)
Satisfaction with function			
Daily activity			
0 weeks	11 (39)	8 (33)	0.78
12 weeks	17 (61)	14 (58)	> 0.9999
*P* (time)	0.18	0.15	
Sport and physical activity			
0 weeks	1 (4)	0 (0)	> 0.9999
12 weeks	7 (25)	4 (17)	0.52
*P* (time)	0.05	0.11	
Treatment satisfaction			
12 weeks	26 (93)	23 (96)	> 0.9999
GROC			
12 weeks	26 (93)	19 (79)	0.23

*Note:* Values are presented as least *n* (%). Chi‐square (and Fisher's exact) test was performed for all analysis based on a contingency table. Alpha level set at *p* < 0.05.

Abbreviations: ER, Extended restitution group; GROC, Global rating of change; SR, Short restitution group.

### 3.4 Function

For maximal isokinetic peak torque, no significant group (*p* = 0.43) or interaction (*p* = 0.53) effects were detected, but there was a significant increase in mean peak torque over time (*p* < 0.0001) (Table 6) of 17% in the ER group and 19% in the SR group. A significant reduction over time (*p* < 0.0001) was found for pain during strength testing, with no group or interaction effects (Table 3). For jump height in both bilateral CMJ and CMJ on the injured side, there were no interaction, group, or time effects (Table 6), but a significant main effect of time was found for pain (*p* < 0.0001) during CMJ both bilaterally and on the injured side. A significant group effect was found for pain during CMJ on the injured side (*p* = 0.02); however, the detected group effect disappears when adjusted for baseline values (*p* = 0.11), indicating that it represents a baseline difference.

**TABLE 6:** Functional results.

			*P*		
	SR (*n* = 28)	ER (*n* = 24)	Group	Time	Group × Time
Muscle strength/BW, Nm/Kg					
0 weeks	2.2 ± 0.1 (2.0 to 2.4)	2.1 ± 0.1 (1.9 to 2.3)	0.43	< 0.0001	0.53
12 weeks	2.6 ± 0.1 (2.4 to 2.8)	2.5 ± 0.1 (2.2 to2.7)			
CMJ height bilat, cm					
0 weeks	28.6 ± 1.2 (26.1 to 31.0)	30.6 ± 1.6 (27.3 to 34.0)	0.31	0.13	0.79
12 weeks	27.5 ± 1.1 (25.2 to 29.8)	29.7 ± 1.7 (26.1 to 33.3)			
CMJ height injured site, cm					
0 weeks	11.5 ± 0.7 (10.1 to 13.0)	12.1 ± 4.4 (9.9 to 14.3)	0.74	0.58	0.54
12 weeks	11.7 ± 0.7 (10.2 to 13.1)	12.2 ± 1.0 (10.1 to 14.2)			

*Note:* Values are presented as least mean ± SEM (95% CI). Mixed effect model was performed for all analysis with time and group as main factors. Alpha level set at *P* < 0.05.

Abbreviations: BW, Body weight; CMJ, Counter movement jump; ER, Extended restitution group; SR, Short restitution group.

### 3.5 Ultrasonography

There was no significant effect of time (*p* = 0.22), group (*p* = 0.19) or interaction (*p* = 0.27) for tendon thickness. Similarly, for PD area there was no group (*p* = 0.38), time (*p* = 0.06) or interaction (*p* = 0.39) effect (Table 7).

**TABLE 7:** Ultrasonography findings injured leg.

				*P*	
	SR (*n* = 28)	ER (*n* = 24)	Group	Time	Group × Time
Power Doppler area, mm^2^					
0 weeks	32.6 ± 6.1 (20.1–45.1)	37.9 ± 6.6 (24.3, 51.6)	0.38	0.06	0.39
12 weeks	24.5 ± 6.3 (11.5, 37.4)	34.9 ± 6.5 (21.5, 48.3)			
Tendon Thickness, mm					
0 weeks	6.8 ± 0.4 (6.1, 7.5)	7.6 ± 0.3 (6.9, 8.3)	0.19	0.22	0.27
12 weeks	6.8 ± 0.5 (5.9, 7.7)	7.3 ± 0.3 (6.7, 8.0)			

*Note:* Values are presented as least mean ± SEM (95% CI). Mixed effect model was performed for all analysis with time and group as main factors. Alpha level set at *p* < 0.05.

Abbreviations: ER, Extended restitution group; SR, Short restitution group.

## 4 Discussion

The present randomized controlled trial investigated if the restitution time from loading in a 12‐week exercise‐based rehabilitation regime for patellar tendinopathy influences the clinical outcome, function, and tendon structure. The data suggests that extended restitution with exercise 1 day per week (ER) was not superior to short restitution with exercise 3 days per week (SR) for the measured clinical, functional, or structural outcome. However, both ER and SR that also included restriction of impact loading yielded significant clinical improvement in the VISA‐P score, pain during physical activity, daily activity, and SLDS. In addition, both groups also improved in muscle strength after 12 weeks, although this did not translate to improvements in jumping ability or tendon structure (thickness or PD area).

Loading‐based rehabilitation has been demonstrated to provide clinical improvements in VISA‐P score in several studies [13, 16, 29]. Likewise, in the present study the VISA‐P score improved over the 12 weeks intervention period by 12.0 (SR) and 14.4 (ER) points, which is in the range of a clinically meaningful change of 13 points [25]. The psychometric properties of the VISA‐P questionnaire as a single score have come into question [26]. Nevertheless, this questionnaire was chosen when designing the current study because it is the only diagnose‐specific questionnaire. A truncated version including only items 2–6 has been suggested to have superior psychometric properties [26], and the present data show that the truncated VISA‐P score also improved significantly over the 12 weeks intervention period. Notably, both groups did not reach 50 points corresponding to the maximal score on the truncated VISA‐P even though items around sport and competition participation (activities that were not allowed during the intervention period) are excluded in the truncated version. In agreement with existing literature, we also observed persistent pain on NRS at week 12 [6], which together with the truncated VISA‐P score supports a lack of full recovery, beyond the limited return‐to‐sport that would contribute to a deficit on the full VISA‐P.

Knowledge about the impact of specific loading parameters in rehabilitation is scarce even though loading‐based treatment is currently the preferred choice for patellar tendinopathy [6]. A recent systematic review [7] suggested that—when resistance exercise is applied—intensity and time for recovery might be important factors to consider. However, we have recently shown that high load magnitude was not superior to moderate load magnitude in relation to clinical outcome, the tendon structure, and function in patellar tendinopathy [16]. The importance of restitution for muscle tissue between training sessions is a well‐accepted concept but has not received any attention in rehabilitation literature in relation to tendinopathies. In tendon cells, a metabolic response to loading has been shown [18, 30] but it is unknown whether rest is required for the purpose of restoring energy deposits. The anabolic response to loading might be sustained in tendon up to several days following an exercise bout [19, 31], indicating that a post‐exercise resting period is an important factor for tendon adaptation [20]. However, contrary to our hypothesis, the current study did not demonstrate a superior effect of extended restitution (ER) compared with short restitution (SR), indicating that more than 1 day of rest between training sessions is not advantageous. The expectation of an enhanced anabolic response was based on previous findings in healthy tendons [19]. Therefore, alterations in tissue composition due to pathology [32, 33] may have influenced the observed outcomes. It is also possible that a single day of rest is enough for the establishment of a net balance synthesis between portion synthesis and degradation following an exercise bout. Importantly, prior investigation of the tissue response to loading has until now only focused on the synthesis side of the response, and very little is known about human tendon protein breakdown [34]. It could therefore be speculated whether altered breakdown camouflages an altered synthesis in response to training in the tendinopathic tissue. Finally, the fact that during inactivity portion synthesis in healthy tendons goes to almost zero [35] indicates that exercise only once a week could have resulted in decreased healing of the tissue in the ER group. However, the comparable improvement in the two groups indicates that load due to daily activity might be sufficient for a certain constant tissue turnover and suggests that stimulating the tendon once per week in combination with unloading from impact activities is sufficient for a clinical improvement. Considered that there is no group difference in scores of improvements and satisfaction with treatment, indicates that the exercise regime can be chosen on basis of patient preferences.

We found no significant change from baseline to week 12 in tendon structure; however, there was a trend for a reduction in the PD area, primarily in the SR group. The lack of change in tendon thickness was expected since it has been shown that 3–4 years after loading‐based treatment the tendon remained thickened [28]. While physical activity is known to influence the vascularization of the healthy and tendinopathic tendon [36], it seems reasonable to assume that restricted impact activity during the intervention period would have contributed to a larger reduction in PD area compared to previous rehabilitation regimes. However, the results from the present study align with previous studies [16, 29, 37] and do not show a larger decrease in doppler activity despite reduced activity level and high compliance with the load‐reduction regime. Notably, a previous study has shown a 45% reduction in PD in response to 12 weeks of heavy slow resistance training [13], which far exceeds that of the current study (SR, 24%; ER, 8%). However, this apparent discrepancy may be related to the tendon biopsies obtained in the other study, as tendon biopsy has been shown to result in increased tendon cell activity. Changes in stiffness have been suggested as another factor influencing symptom improvement in Achilles tendinopathy [38]. However, in tendinopathic patellar tendons, both unchanged [13, 39] and decreases [40, 41] mechanical stiffness in response to training have been observed, independent of clinical improvements.

It is well accepted that greater resistance training frequency results in more strength gain in healthy individuals, which seems to be driven by training volume [42]. However, in the current study both groups gained equally in strength (SR, 21%; ER, 20%) despite the difference in training volume. A possible explanation for this finding could be that pain is the limiting factor in the actual strength test and training load. In fact, the participants did not improve their jumping ability, supporting that notion that the gain in strength is limited and not large enough to transfer to improvement in jump height. However, pain or fear of pain could potentially also have influenced the jumping ability.

This study has inherent limitations. A non‐exercise control group was not included, and therefore, it cannot be ruled out that improvements would have occurred over time in the absence of the loading regime; however, the chronic status of the patients suggests that wait‐and‐see would not be sufficient for recovery. Further, the study was designed to test the influence of restitution time; however, due to the interaction of parameters in the loading regime, the total exercise volume also differed between the groups, while load, sets, and repetitions per exercise were the same. If volume should have been kept constant, it would instead require a difference in the number of sets performed, and we considered 9–12 sets on 1 day to risk overloading the tendon. Additionally, measures of activity, including high‐impact loading, are based on patient reports rather than objective activity measurements. However, due to the randomized study design, this is expected to influence the groups equally.

## 5 Conclusion

In conclusion, the current study demonstrated that there was no statistically superior effect of extended restitution (exercise 1 day per week) compared to short restitution (exercise 3 days per week) when impact activities were restricted in both groups. Both groups demonstrated comparable clinical improvement after 12 weeks, but no significant improvement in tendon structure (thickness or PD area). Moreover, there were no group differences in self‐reported improvement or satisfaction with function or treatment at week 12. The absence of group differences in improvement and treatment satisfaction scores suggests that it is possible to select the exercise regime based on patient preferences.

### 5.1 Perspective

There has been considerable focus on the clinical effects of loading‐based exercise regimes as treatment for tendinopathy, but very little is known about the importance of changing specific loading parameters [43]. When designing an exercise intervention, key parameters include the load magnitude, duration, speed, and restitution time. The present study showed that restitution time between loading exposure did not influence the clinical outcome, tendon function, or tissue response of tendinopathic tendons. Clinical outcome and muscular strength improved after 12 weeks intervention, showing that restitution time may be tailored to the patient, although outcomes did not reach normal values regardless of restitution time. Conversely, 12 weeks was not sufficient for improvement in the structural outcome.

## Funding

This work was supported by Lundbeck Foundation. Bispebjerg and Frederiksberg Hospital. Danish Society of Physiotherapy. Danish Society of Sports Physiotherapy.

## Ethics Statement

Ethical approval was obtained from the regional Ethics Committees for medical research (H‐22031880) and all participants provided written informed consent.

## Conflicts of Interest

The authors declare no conflicts of interest.

## Supporting information


**Appendix S1:** sms70235‐sup‐0001‐AppendixS1.pdf.

**Appendix S2:** sms70235‐sup‐0002‐AppendixS2.pdf.

**Table S1:** Baselines characteristics.
**Table S2:** Clinical Results.
**Table S3:** Sports participation (hr/wk).
**Table S4:** Satisfaction and Improvement.
**Table S5:** Functional results.
**Table S6:** Ultrasonography findings injured leg.
**Figure S1:** Clinical improvement in VISA‐P. Values are presented as mean ± SEM at baseline (0wk) and after intervention (12wk) for the two intervention groups. VISA‐P, Victorian Institute of Sports Assessment–Patella, SR, Short restitution group; ER, Extended restitution group. Mixed effect model was performed for all analysis with time and group as main factors. Alpha level set at p < 0.05. P‐values group (0.16), time (< 0.0001), and interaction (0.54).
**Figure S2:** Figures showing individual participant data for VISA‐P, VISA‐P truncated, NRS SLDS, and NRS Daily activity. VISA‐P, Victorian Institute of Sports Assessment–Patella; SLDS, Single leg decline squat; Short restitution group; ER, Extended restitution group.

## Data Availability

The data that support the findings of this study are available from the corresponding author upon reasonable request.
