# Supplementary material for: Extended Restitution Between Sessions Does Not Enhance the Benefits of 12 Weeks Exercise‐Based Treatment for Patellar Tendinopathy: A Randomized Controlled Clinical Trial (The TEREX Trial)
Source: Scand J Med Sci Sports. 2026 Mar 8;36(3):e70235. doi: 10.1111/sms.70235 (PMC12968374; doi:10.1111/sms.70235)
Supplement: Supplementary file 1 — Appendix S1: sms70235‐sup‐0001‐AppendixS1.pdf. [file SMS-36-e70235-s003.pdf]

MANUAL FOR THE EXERCISE AND LOAD REDUCTION INTERVENTION IN THE TEREX TRIAL - PHASE ONE

1. Brief name

Exercise therapy with different restitution and load reduction

2. Why

This document describes the details of the exercise program and the load reduction strategi and will be used for training of intervention providers in the TEREX trial phase one.

In recent years the dominant conservative treatment strategy for tendinopathy has become exercise-based interventions [1,3]. Different loading regimes have been applied, however we recently showed that high load magnitude was not superior compared to a moderate load magnitude with the same total exercise volume. Importantly, both loading regimes resulted in clinical improvements at both the short and long term, but the patients did not achieve complete recovery. This study, however, was designed to strictly test the influence of load magnitude alone. The impact of other exercising variables, such as restitution time, on the outcome and lack of full recovery remains to be determined.

Firstly, the overall goal of the intervention is by slow velocity loading-based exercises to provide controlled mechanical load to the patellar tendon to promote remodeling and thereby improve tendon structure and function, and decrease pain. Secondly, a goal of the intervention is to further increase the effect and to achieve full recovery by increasing the restitution between loading sessions. The rational for this component of the intervention is that net balance between synthesis and degradation of collagen in response to a single bout of loading has been suggested to be negative up to 36 hours after exercising with positive synthesis lasting from 36 to 72 hours [2]. Thirdly, to ensure sufficient tendon recovery between training session, patients in the present study will have complete cessation of impact activity with high tendon loading (jumping and running) during the first 12 weeks of the intervention. A recent pilot trial, have shown that the use of pain-guided activity modification for athletes with patellar tendinopathy (PT) appears feasible[4] but a caveat to reducing the frequency of controlled exercises is that it leaves more time for other uncontrolled exercise activities. This could negate the effects of restitution and potentially exacerbate the tendinopathy. Therefore, to ensure sufficient tendon recovery between training sessions, patients in the present study will have complete cessation of impact activity with high tendon loading (jumping and running) during the first 12 weeks of the intervention. In addition, the pain-monitoring model will be used to guide exercise progression and keep the amount of non-impact activity at an acceptable level during the intervention period. Finally, supervision will be included to support quality and safe progression of the intervention and further support compliance to the allocated intervention and enable patient education in adjusting activity level.

3. What

3.1 Materials

The exercise therapy will require access to strength training equipment with different resistance, and all participants will be given access to a commercial fitness center.
All participants will receive practical information and information on the exercise program, load progression and allowed pain in a written pamphlet (Appendix 1 – 'TEREX studiet del 1: Informationspjece for patienter'). The information about the intervention will also be available in the electronic training diary. Furthermore, they will receive a pamphlet with suggestions of non-impact activities that can be used as inspiration during the period with load reduction and activity modification (Appendix 2 – TEREX studiet: Guide til aktiv aflastning). A detailed written information (Appendix 3 – TEREX studiet del 1: Guide for intervention providers) including description of how the intervention is carried out, focus point for quality check, adjustments opportunities and how to perform load progression will be used in training of and as a working tool for the intervention providers.

3.2 Procedures

This exercise program runs for 12 weeks with exercise sessions 1 or 3 times per week. Each training session is scheduled to last approximately 60 minutes. The exercise is facility based and will be performed individually in a commercial fitness center. One weekly session in weeks 1,3 and 6 will be carried out under supervision of a physiotherapist and the remaining sessions will not be supervised. However, the participants will receive a text in week 2 and 4 and a phone call during week 9 from the physiotherapist. The intervention program includes exercise therapy and load reduction from impact activities as running and jumping. Moreover, compliance with exercise and activity modification will be tracked using a training diary.
Focus of the exercises is on the quality of the performance – not quantity. Each exercise has a specific focus as specified in the exercise description below and moreover, an overall focus is on maintaining slow and controlled motion during the entire course of the program. This is emphasised to the participants during the supervised sessions.

Load reduction

Participants in both programs are not allowed to performed impact activities (running and jumping activities) that may provoke their patellar tendon outside of the prescribed treatment. Likewise, participants will be guided by the supervising physiotherapist in how to avoid strength training involving the quadriceps muscle. However, they will be encouraged and guided to perform non-impact activities (e.g. biking, swimming, rowing, strength training not involving the quadriceps muscle and any part of their normal training not including impact on the patellar tendon).

Compliance

Compliance with exercise and activity modification will be tracked using a training diary. Participants will be asked to record the number of sessions and load of the treatment exercises completed and whether they performed running, jumping or other activities outside of the intervention.

The patient will be defined as 'compliant' with the treatment if they have performed at least 80% of the prescribed exercise sessions. I addition, patients will only be defined as 'compliant' if they complied have complied with the load-reduction for at least 10 of the 12 weeks.

The exercise program for the short restitution (SR) and extended restitution (ER) group
The exercise program described below will be identical for both groups apart from the short restitution (SR) group performing the resistance exercise three times per week and the extended restitution (ER) group only once per week (increased restitution time). For the SR-group there must be at least 48 hours rest and for the ER-group 96 hours between each training session.

The program will be started at a load of 15RM (~60% of 1 RM) and progressed to 10RM (~75% of 1 RM) during the first 3 weeks and maintained throughout the intervention. The volume is 3 or 4 sets in each exercise with 1-2 minutes of rest between sets. Information about the loading protocol is summarized in Table 1.

**Exercise protocol**				
**Week**	**1**	**2-3**	**4-5**	**6-12**
**Sets and reps**	3 x15	3 x 12	3x 10	4 x 10
**~ % of 1 RM**	60	70	75	75

Table 1: Loading protocol, identical for the two groups

All patients will be instructed to perform the exercises with a slow velocity, spending three seconds completing each of the eccentric and concentric phases, respectively (i.e 6 s/repetition).

The exact trainings load for each exercise is estimated based on the repetition maximum (RM) listed in table 1. Each exercise is initiated with loading that achieves muscle fatigue within 15 repetition in each set. An increase in load is recommended when the participants is able to perform 2 repetitions more than the desired number (i.e. 17 or more) with satisfying quality and within the limits of pain.

Warm up
Participants are instructed to warm-up by 5-minute cycling on a stationary bike ergometer with moderate intensity (corresponding to 11-15 on Borg Rating of Perceived Exertion Scale; “somewhat hard”).

Exercises

LEG PRESS – Bilateral

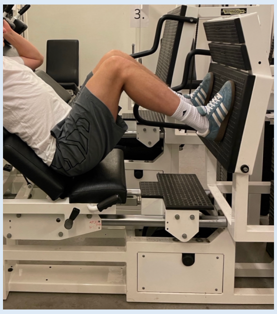 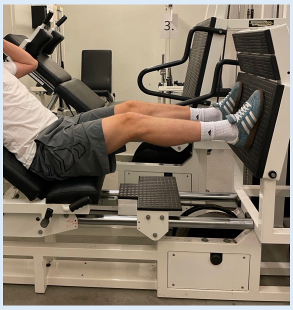

Purpose:

- To provide mechanical load to the patellar tendon to promote remodeling of the tissue and thereof decrease pain, and improve the leg function.

Exercise description:

- Select the required amount of weight.
- Start out sitting on a leg press machine with both feet shoulder-width apart against the footplate and approximately 90° of knee flexion.
- Grasp the handle grips and keep your entire back firmly set against the seat for stability during the exercise.
- Push the weight with both legs equally, to a knee flexion angle of 10° and then slowly bring the weight back to the starting position.
- Perform the exercises in a slow and controlled fashion, spending three seconds completing each of the eccentric and concentric phases, respectively (i.e 6 s/repetition).
- Perform the number of repetitions and sets according to the week-number of the intervention program, with 1-2 minutes of rest between sets.

Focus:

Main focus is on keeping the slow velocity and the recommended rest between the set. Further, when increasing the amount of load focus on the position of the back and make sure the pelvis is not rotated. In the end range of knee extension, making sure the movement is ended in 10° of knee flexion.

Progression:

Load progression will be individually adjusted as detailed in *table 1*. The patients will be instructed that the load magnitude has to be high enough that they are only just able to perform the established repetitions pr set within the limit of pain/ exhaustion. If more repetitions can be performed, the load is increased in subsequent sets.

Regression:

If the participant is not able to perform the scheduled number of sets or repetitions due to fatigue or pain in the tendon, firstly ensure that the exercises are performed in slow action.
Otherwise, load will be adjusted due to patient self-reported pain. Pain during treatment exercises is accepted if 5 or below on the NRS, but pain and discomfort should not increase following cessation of training and if any training-induced pain did not subside 3 to 4 hours after the session, the load will be reduced during the next session.

LEG EXTENSION – Unilateral

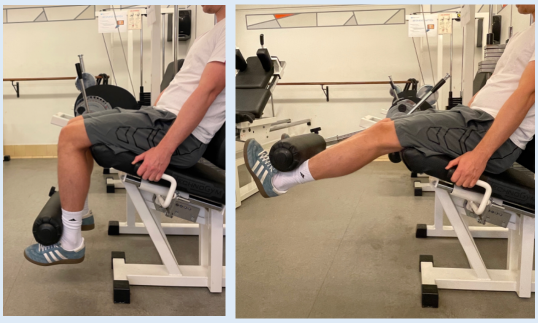

Purpose:

- To provide mechanical load to the patellar tendon to promote remodeling and thereof decrease pain, and improve the leg function.

Exercise description:

- Select the required amount of weight.
- Start out sitting on a leg extension machine with one leg under the padded bar and approximately 100° of knee flexion. Leave the contralateral leg resting during the exercise.
- Grasp the handle grips and keep your entire back firmly set against the seat for stability during the exercise.
- Push the weight with one leg, to a flexion angle of 10° and then slowly bring the weight back to the starting position.
- Perform the exercises in a slow and controlled fashion, spending three seconds completing each of the eccentric and concentric phases, respectively (i.e 6 s/repetition).
- Perform the number of repetitions and sets according to the week-number of the intervention program.

Focus:

Main focus is on keeping the slow velocity and the recommended rest between the set. Further, when increasing the amount of load focus on the position of the back and making sure the pelvis and the foot is not rotated. In the end range of knee extension, making sure the movement is ended in 30° of knee flexion.

Progression:

Load progression will be individually adjusted based on the load progression in *table 1*. The patients will be instructed that the load magnitude has to be high enough that they are only just able to perform the established repetitions pr set. If more repetitions can be performed, the load is increased in subsequent sets.

Regression:

If the participant is not able to perform the scheduled number of sets or repetitions due to fatigue or pain in the tendon, firstly ensure that the exercises are performed in slow action.
Otherwise, load will be adjusted due to patient self-reported pain. Pain during treatment exercises is accepted if 5 or below on the NRS but pain and discomfort should not increase following cessation of training and if any training-induced pain did not subside 3 to 4 hours after the session, the load will be reduced during the next session.

RESCUE EXERCISE PROGRAM
If a participants scores knee pain above 5 on a 0-10 NRS scale when attending for an exercise session, he/she is transferred to the rescue program.<br><br>The recure program is a modified program consisting of an extended warm up (15 minutes) and return to the load, set and reps from the latest training session with acceptable pain.<br><br>The program will continue until there is no longer flare-up of symptoms.

5. Who provide

Physical therapists with experience of training recreational athletes (intervention providers) will introduce the participant to the exercise program and deliver the supervision during the intervention period.

The intervention providers will participate in a training session before initiation of the project. In this session, the primary investigator (clinical experience as a sports-physiotherapist, working with patients with patellar tendinopathy) will introduce the intervention providers to the written material and practically introduce the exercises and how to progress, regress and correctly.

During the intervention period, it will be possible for the intervention providers to have ad hoc supervision and professional back-and-forth with the responsible of the project.

6. How

The exercise is facility based and will be performed individually in a commercial fitness center. One session in weeks 1, 3 and 6 will be carried out under supervision of a physiotherapist one patient at a time, and delivered face to face. In week 2 and 4 the patient will be remined of changing the training load by a text message and in week 9 the physiotherapy will follow-up on the training intervention, compliance and to what extent complied with the load-reduction with a phone-call.

7. Where

The unsupervised sessions will be performed in a commercial fitness center. The setting of training in a commercial fitness center is chosen as it matches how this kind of intervention is performed in clinical practice.
The supervised session will take place at the physiotherapy department at Bispebjerg Hospital, and is identical with usual care of patient with patellar tendinopati at the department.

8. When and how much

The exercise program described below will be identical for both groups apart from the SR-group performing the resistance exercise three times per week and the ER-group only once per week (increased restitution time). For the SR-group there must be at least 48 hours rest and for the ER-group 96 hours between each training session.

Each session will consist of two exercises, leg press (bilateral) and knee extension (unilateral) performed in slow action (i.e 6 s/repetition). Participants will be instructed to perform three or four sets in each exercise with a 1-2 min rest between sets. The loading program will be started at 60% of 1 RM and progressed to 75% of 1 RM during the first 3 weeks and maintained throughout the intervention period.

9. Tailoring

The load in each exercise will be tailored to the individual patient based upon individual strength. Moreover, load will be adjusted due to patient self-reported pain on a NRS scale.

Knee pain monitoring

A 0-10 numeric rating Scale (NRS, 0 equals no pain and 10 equals worst imaginable pain, figure 1) is used by the participant to assess their patellar tendon pain before, during and after each training session.
Pain during treatment exercises is accepted to reach 5 on the NRS (yellow /orange area) but pain and discomfort should not increase following cessation of training and if any training-induced pain did not subside 3 to 4 hours after the session, the load will be reduced during the next session. The pain ratings are recorded in the participants training diary at each session.

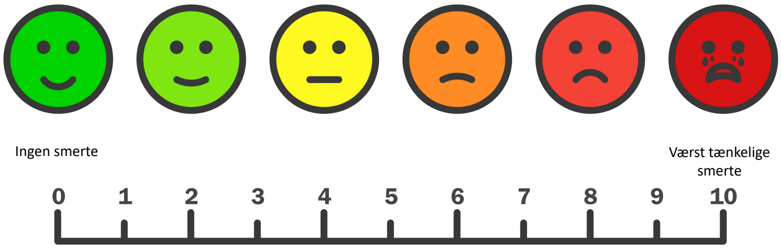

Figure 1: Visual numeric rating scale for pain monitoring ranging from 0 (no pain) to 10 (worst imaginable pain).

Both groups are instructed to only perform other non-impact activities if patellar tendon pain can be maintained below 3 (green area) or more on the 0-10 NRS. If pain intensity exceeds 3 on the NRS, the participant is recommended to stop the activity.

References

1. Larsson MEH, Käll I, Nilsson-Helander K. Treatment of patellar tendinopathy-a systematic review of randomized controlled trials. *Knee Surgery, Sport Traumatol Arthrosc*. 2012;20(8):1632-1646. doi:10.1007/s00167-011-1825-1.
2. Magnusson SP, Langberg H, Kjaer M. The pathogenesis of tendinopathy: Balancing the response to loading. *Nat Rev Rheumatol*. 2010;6(5):262-268. doi:10.1038/nrrheum.2010.43.
3. Malliaras P, Barton CJ, Reeves ND, Langberg H. Achilles and Patellar Tendinopathy Loading Programmes. *Sport Med*. 2013;43(4):267-286. doi:10.1007/s40279-013-0019-z.
4. Sprague AL, Couppé C, Pohlig RT, Snyder-Mackler L, Silbernagel KG. Pain-guided activity modification during treatment for patellar tendinopathy: a feasibility and pilot randomized clinical trial. *Pilot Feasibility Stud*. 2021;7(1):1-17. doi:10.1186/s40814-021-00792-5.

Bispebjerg Hospital

Fysio- og
Ergoterapiafdelingen

TEREX studiet del 1: Informationspjece for patienter

Praktisk
Du er ved tilfældig lodtrækning tildelt genoptræningsgruppen med; *kort restitution og skal derfor træne nedenstående program 3 gange om ugen/ lang restitution og skal derfor træne nedenstående program 1 gang om ugen.*

Træningsprogrammet i TEREX studiet del 1 forløber over 12 uger. Nogle vil opleve bedring allerede efter de 12 uger, og andre først efter flere måneders træning evt. suppleret med anden behandling. Du må have tålmodighed og vedholdenhed for, at det skal lykkes.
Efter de 12 ugers træning vil du blive bedt om at vurdere effekten af behandlingen og på baggrund heraf udarbejdes i samråd med den projektansvarlige en plan for dit videre behandlingsforløb under TEREX studiets del 2.

Øvelserne i programmet kræver adgang til et træningscenter. Du blive givet adgang til træning i SATS og vil inden den aftalte startdato modtage yderligere information herom pr mail.

I din træningsuge 1, 3 og 6 vil én træningssession om ugen være superviseret af en idrætsfysioterapeut. Supervisionen vil foregå i fysioterapien på Bispebjerg Hospital. Dato og tid for de enkelte supervisionsgange aftales med den superviserende fysioterapeut.

Du vil løbende modtage en elektronisk dagbog på mail via REDCap. Det er meget vigtigt for projektet at du husker at udfylde denne træningsdagbog.

Anden aktivitet
I denne første del af projektet (12 uger) laves styrketræning af musklen på forsiden af låret med det formål at nedsætte smerten og fremme heling af senevævet samtidig med at knæskalssenen aflastes. På dette stadie, skal du

derfor undgå løbe- og hoppeaktiviteter og anden aktivitet der resulterer i smerter udover det grønne område på smerteskalaen beskrevet nedenfor.
Du bør dog så vidt muligt forblive fysisk aktiv med andre aktiviteter og den superviserende fysioterapeut vil derfor vejlede dig i at tilpasse din belastning (aktiv aflastning).

Lad smerterne guide dig
Smerteskalaen kan være en hjælp, når du skal vurdere om en aktivitet er tilpas eller for meget.

Når du laver **trænings øvelser i projektet**, må du gerne have ondt op til det gule område på smerteskalaen. Du skal dog opleve at smerten hurtigt fortager sig efter endt træning og ikke er forværret dagen efter.

For **andre aktiviteter** gælder det at, hvis du kan være aktiv og kun have smerte svarende til det grønne område på skalaen, så kan du fortsætte. Hvis du kommer ind i det gule/orange område, skal du være mere opmærksom, og måske overveje at begrænse eller stoppe aktiviteten. Oplever du smerter i det røde felt, skal du undgå aktiviteten i en periode.
Pointen er, at du gerne må lave de aktiviteter, hvor smerten ikke påvirker dig særligt meget, og hvor den kendte smerte i knæet ikke er forværret næste morgenen eller i dagene efter. Du må gerne være øm i musklerne, da det er et tegn på, at du har trænet dem.

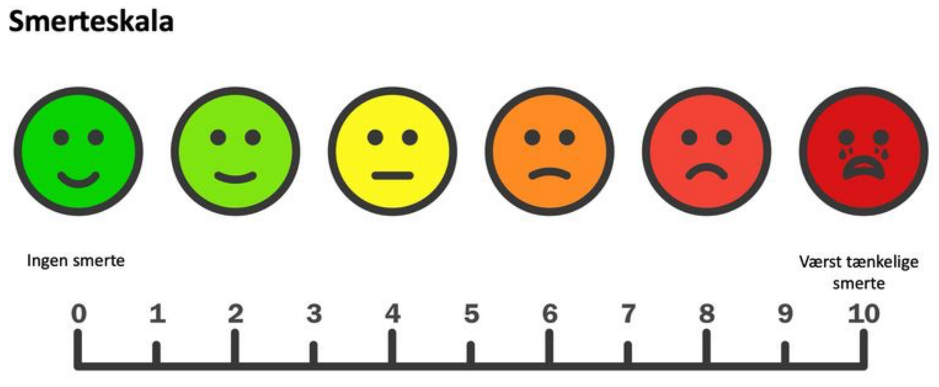

Træningsprogram
Øvelsesprogrammet består af 5 minutters opvarmning på kondicykel og nedenstående to øvelser. Benpres udføres med begge ben på en gang, mens

knæ ekstension trænes med et ben af gangen.

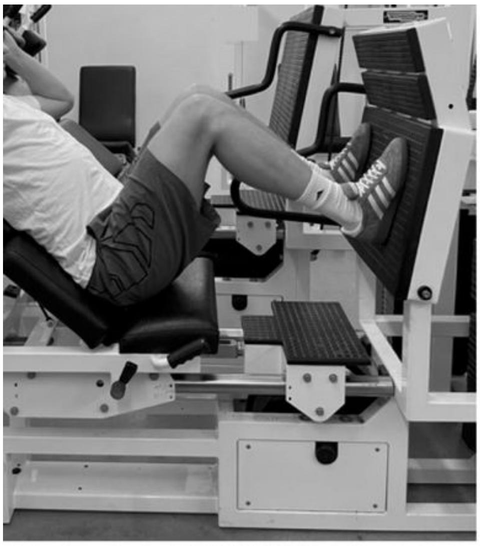

BENPRES

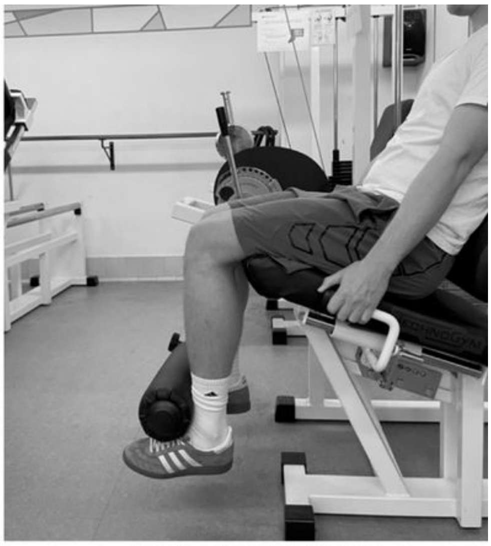

KNÆ EKSTENSION

Øvelserne trænes ***3/1*** gange om ugen (mindst ***48/96*** timers pause mellem hvert træningspas).
Belastningen progredieres som i tabellen. I eksempelvis første uge skal du altså træne 3 sæt á 15 gentagelser for hver øvelse med 1-2 minutters pause mellem hvert sæt.

Træningsprotokol

Uge	1	2-3	4-5	6-12
Sæt og gentagelser	3x15	3x12	3x10	4x10

Øvelserne skal udføres **meget langsomt**. Hver gentagelse skal tage 6-8 sekunder (3-4 sekunder hver vej).

Din superviserende fysioterapeut vil instruere og guide dig i korrekt udførelse og progression af træningsprogrammet samt følge op på din træningsdagbog.

Appendix B

TEREX studiet: Guide til aktiv aflastning

Springerknæ er en overbelastningsskade af knæskalssenen. Skaden opstår ved for hyppig og for stor belastning af lårmusklen og dermed knæskalssenen. Du er derfor blevet bedt om at **undgå løbe og hoppe aktiviteter** i de første **12 uger** af genoptræningsperioden.

Du bør så vidt muligt forblive fysisk aktiv med andre aktiviteter der ikke overbelaster din knæskalssene og/ eller provokerer dine smerter.

Lad smerterne guide dig
Smerteskalaen herunder kan være en hjælp, når du skal vurdere om en aktivitet er tilpas eller for meget. Hvis du kan være aktiv og kun have smerte svarende til det grønne område på skalaen, så kan du fortsætte. Hvis du kommer ind i det gule/orange område, skal du være mere opmærksom, og måske overveje at begrænse eller stoppe aktiviteten. Oplever du smerter i det røde felt, skal du undgå aktiviteten i en periode.
Pointen er, at du gerne må lave de aktiviteter, hvor smerten ikke påvirker dig særligt meget, og hvor den kendte smerte i knæet ikke er forværret om morgenen eller i dagene efter. Du må gerne være øm i musklerne, da det er et tegn på, at du har trænet dem.

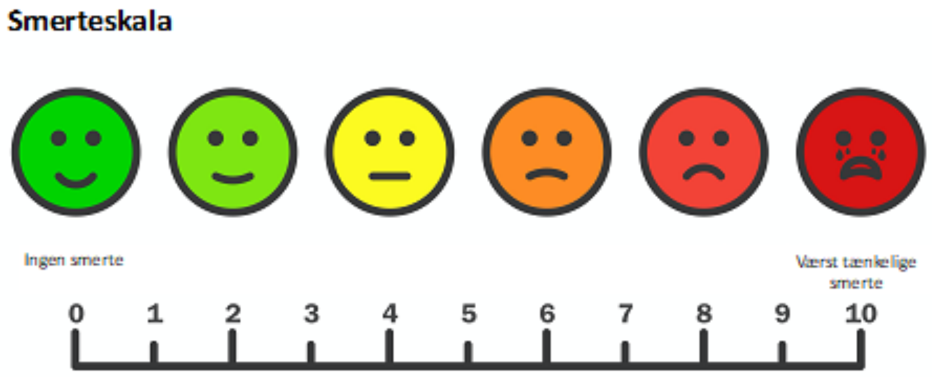

Alternative aktiviteter til at vedligeholde din kondition kunne være:

- Cykling/ kondicykling
- Crosstrainer
- Romaskine
- Svømning
- Løb i vand

Ofte vil det også være muligt fortsat at deltage i dele af din almindelige træning f.eks.:

- Teknisk træning
- Kasteøvelser
- Styrketræning der ikke inkludere lårmusklen.
- Stabilitetstræning
