# Supplementary material for: Extended Restitution Between Sessions Does Not Enhance the Benefits of 12 Weeks Exercise‐Based Treatment for Patellar Tendinopathy: A Randomized Controlled Clinical Trial (The TEREX Trial)
Source: Scand J Med Sci Sports. 2026 Mar 8;36(3):e70235. doi: 10.1111/sms.70235 (PMC12968374; doi:10.1111/sms.70235)
Supplement: Supplementary file 2 — Appendix S2: sms70235‐sup‐0002‐AppendixS2.pdf. [file SMS-36-e70235-s002.pdf]

Clinical study protocol:

Patellar tendinopathy - The role of restitution time in exercise- based treatment: A Randomized controlled trial (the TEREX trial)

(*Danish title:* Betydning af restitution i behandling af Springerknæ: Et randomiseret kontrolleret forsøg (TEREX studiet))

Responsible and principal Investigator & Contact Person:
Anne-Sofie Agergaard, Post doc, PhD, PT, Institut for Idrætsmedicin og Fysio- og Ergoterapiafdelingen, Bispebjerg Frederiksberg Universitets Hospital
Tlf.: 38635675
E-mail: anne-sofie.agergaard@regionh.dk

Institution:
Institut for Idrætsmedicin og Fysio- og Ergoterapiafdelingen,
Bispebjerg Frederiksberg Universitets Hospital
Nielsine Nielsens Vej 11, Indgang 8, 1. sal
2400 København NV

Table of Contents

1.0 ADMINISTRATIVE INFORMATION 4

1.1 Title 4
1.2 Trial registration 4
1.3 Protocol version 4
1.4 Funding 5
1.5 Roles and responsibilities 5

2.0 INTRODUCTION 6

3.0 BACKGROUND 6

4.0 STUDY AIM, HYPOTHESIS AND OBJECTIVES 9

4.1 Aim 9
4.2 Hypotheses 9
4.3 Objectives 9

5.0 STUDY DESIGN 10

5.1 Trial design 10
5.2 Study setting 12
5.3 Patient involvement 12
5.4 Study design considerations 12

6.0 PARTICIPANTS 13

6.1 Eligibility criteria 13
6.2 Diagnosis under study 14
6.3 Selection of study tendon 14
6.4 Allocation of participants and sequence generation 14
6.5 Blinding 15
6.6 Sample size 15
6.7 Participants recruitment 16

7.0 STUDY INTERVENTION 16

7.1 Phase one (Main trial) - Exercise therapy with different restitution and load reduction 16
7.2 Phase 2 – Follow-up treatment in the main study 18
7.3 Sub-study two – Add on treatment of injection, exercise therapy and load reduction 20

8.0 OUTCOME ASSESSMENT VARIABLES 20

8.1 Primary Outcome 20
8.2 Secondary outcome 20
8.3 Other outcomes 21

9.0 DESCRIPTION OF THE OUTCOME 21

9.1 Clinical picture 21
9.2 Functional test 22
9.3 Imaging technologies 22
9.4 The Pressure pain threshold 23

10.0 STUDY PROCEDURE 23

10.1 First contact 23
10.2 Assessment schedule 23
10.3 Participants time requirements 25
10.5 Treatment visit window 26

10.4 Assessment window 27
10.5 Treatment visit window 27
10.6 Time plan for recruiting patients and testing 27
10.7 Logistics 27

11.0 DISCONTINUATION 27

11.1 Participant withdrawal 27
11.2 Individual participant discontinuation 28
11.3 Discontinuation of Entire Study 28

12.0 STATISTICAL METHODS 28

13.0 ETHICAL ASPECTS, RISKS AND SIDE EFFECTS 29

13.1 Research ethic intervention and assessment 29
13.2 Risks contra benefits of this study 30

14.0 REGULATORY STANDARDS 30

14.1 Storage of data 30
14.2 Patient insurance 31
14.3 Roles and responsibilities 31
14.4 Funding 31

15.0 PUBLICATION OF RESULTS 31

16.0 COMPLETION OF THE STUDY 32

17.0 REFERENCES 33

1.0 Administrative information

1.1 Title

Full title of trial

Patellar **TE**ndinopathy - The role of **R**estitution time in **EX**ercise- based treatment: A Randomized controlled trial (the TEREX trial)

Acronym

The TEREX trial

Short title

Influence of restitution time in treatment of patellar tendinopathy

1.2 Trial registration

Health Research Ethics Committee Number:

Pending

Public data controller at Region H Number

Pending

ClinicalTrials.gov Number

The study will be registered by ClinicalTrials.gov prior inclusion of the first patient.

1.3 Protocol version

Data: January 17, 2023

Version: 3.0

Revision history

**Version #**	**Issue date**	**List of major changes**
1.0	25.05.2022	This is first draft
2.0	23.09.2022	Version med rettelser for betinget godkendelse
3.0	23.02.2023	Verion med tilrettet intervention, supervision og outcomemeasures for ’tilægsprotokol 1’

1.4 Funding

The project will be funded by external research foundation grants and the Department of Physical and Occupational Therapy, Bispebjerg Frederiksberg Hospital.

1.5 Roles and responsibilities

Responsible and principal Investigator:

Anne-Sofie Agergaard, Post doc, PT, PhD, Department of Physical and Occupational Therapy/ Institute of Sports Medicine Copenhagen, Bispebjerg Frederiksberg University Hospital.

Responsible clinical investigator:

Finn Johannsen, MD, Institute of Sports Medicine Copenhagen, Bispebjerg Frederiksberg University Hospital

Investigators:

S. Peter Magnusson, Professor, B.Sc., DMSc, Department of Physical and Occupational Therapy / Institute of Sports Medicine Copenhagen, Bispebjerg Frederiksberg University Hospital, Faculty of Health & Medical Sciences, University of Copenhagen.

Rene B. Svensson*,* Senior researcher, PhD, Institute of Sports Medicine Copenhagen, Bispebjerg Frederiksberg University Hospital

Jesper Pedersen, MD, PhD, Institute of Sports Medicine Copenhagen, Bispebjerg Frederiksberg University Hospital

Carl-Johan Boraxbekk, Professor, PhD, Institute of Sports Medicine Copenhagen, Bispebjerg Frederiksberg University Hospital, Danish Research Center for Magnetic Resonance, Hvidovre Hospital, and Department of Clinical Medicine, University of Copenhagen.

Patient partners:

Claus Frederiksen, patellar tendinopathy patient.
Frederik Nobel, patellar tendinopathy patient.

2.0 Introduction

Tendons play an essential role in transmitting force from muscle to bone and are thus designed to resist considerable loads during locomotion[1]. Yet, repetitive use often results in overuse injuries, such as tendinopathy, which is characterized by pain during activity, localized tenderness upon palpation, impaired performance and structural changes like swelling and hypervascularization[2]. Overuse injuries accounts for 30-50% of all physical activity related injuries[3] and the incidence of lower extremity tendinopathies in Danish general practice has been reported to be as high as 7.9 per 1000 registered patients[4]. Notably, this type of injury is a substantial clinical challenge because it often impacts function and work ability long-term (months to years)[5,6], and is consequently a sizeable economic burden on society[7]. Loading based treatment is currently the preferred treatment for tendinopathy, although the optimal loading configuration and time of loading remains unknown[8].

We have previously investigated to what extent load magnitude (high and moderate load) influences the clinical outcome, the tendon structure, and mechanical function in patients with patellar tendinopathy[9]. Importantly, both high and moderate loading regimes yielded similar improvements in all outcome measures in the short term. Moreover, the improvements were maintained in the long term (12 months), however, most of the patients, unfortunately, did not reach normal function even after one year, and the reason for this incomplete recovery remains an enigma. This has prompted questions that relate to whether other exercising variables may improve the treatment of tendinopathy patients. Specifically, the present project aims to answer whether the restitution duration impact the outcome and ability to fully recover from tendinopathy. Potential benefits of increased restitution time are based on basic biological considerations, and may also benefit patient adherence to the program in a "how low we can go" sense, by reducing the amount of time / planning required for the patient.

3.0 Background

Tendinopathies are very common and a substantial clinical challenge because the exact pathologic characteristics and optimal treatment modalities remain elusive[10,11]. The list of currently available treatment modalities for tendinopathy is broad and may include; exercise therapy, surgery, nonsteroidal anti-inflammatory drugs, steroid injection, platelet-rich plasma injection, therapeutic ultrasound, sclerosing therapy and extracorporeal shock wave therapy[12,13]. However, in recent years the dominant conservative treatment strategy for tendinopathy has become exercise-based interventions[8,14].

Since beneficial outcomes of a stretch-shortening exercise program first placed attention on exercise as a treatment of tendinopathy back in the 80´s[15], varying treatment regimens for these challenging injuries, including different loading configurations, have been suggested. Later

eccentric exercise paradigms[16] gained considerable popularity despite a lack of clinical or mechanistic evidence to support isolating the eccentric component[8,17,18]. More recently new loading-based exercise regimes, such as heavy slow resistance training has emerged. Much of the international attention on heavy slow resistance training as loading regime came from a study comparing the effects of corticosteroid injection, eccentric loading, and heavy slow resistance training on the clinical outcome and tissue and biochemical response in patients with patellar tendinopathy in our lab[19]. The results showed that heavy slow resistance training yielded a favorable outcome with respect to the clinical picture (pain and function), structural composition (collagen fibril diameter and fibril density), and biochemical composition (enzymatically derived collagen cross-links), although recovery at the final half-year follow-up remained incomplete. The available evidence from clinical trials[19,20] and from basic science[21–23] suggested that load magnitude favoring heavy loads was an import component in attempting to optimize the treatment response. Based on this we therefore recently investigated[9] to what extent load magnitude during treatment influenced the clinical outcome, the tendon structure, and the function of tendinopathic patellar tendons. The results showed that high load magnitude was not superior compared to a moderate load magnitude with the same total exercise volume. Importantly, both loading regimes resulted in clinical improvements at both the short and long term, but the patients did not achieve complete recovery. *This study, however, was designed to strictly test the influence of load magnitude alone. The impact of other exercising variables, such as restitution time, on the outcome and lack of full recovery remains to be determined.*

It is well known that pain in people with chronic disease might include a centralized component and there is a rising interests in non-invasive magnetic resonance imaging (MRI) as imaging tool for assessment of altered brain structure and pain perception[24]. However, it remains unknown if the same centrally modification occur in response to chronic patellar tendinopathy. Promising MRI with Blood Oxygenation Level Dependent (BOLD) imaging techniques have been explored[25]. While this technique have been used for mapping brain structure, function and metabolism in relation to aging there are currently no studies that have examined the applicability in relation to assessment of brain structure, brain functional connectivity, and brain metabolism in chronic tendinopathy and rehabilitation thereof. *We intend to bridge this gap in knowledge in the current explorative investigation.*

Effects of load restitution on tendon

The importance of restitution for muscle tissue between training sessions is a well-accepted concept. For example, resistance training to achieve increased strength can at times be counterproductive if performed >2 times per week[26]. However, such considerations have yet to be incorporated in rehabilitation of tendinopathies. Our lab has previously shown that tendon cells are metabolically active in response to loading[27], but it is unknown whether rest is required for the purpose of restoring energy deposits considering the relatively low metabolism. On the other

hand, the anabolic response to loading is sustained in tendon up to several days following an exercise bout[28,29], which could indicate the need for a post exercise resting period[30]. Most all eccentric exercise protocols for tendinopathy management are performed every day without rest periods[16,31], however, net balance between synthesis and degradation of collagen in response to a single bout of loading has been suggested to be negative up to 36 hours after exercising with positive synthesis lasting from 36 to 72 hours (figure 1)[30]. *It remains unknown if increased restitution from loading will yield a more positive clinical outcome, structure and function of tendon in patients with patellar tendinopathy, and we intend to bridge this gap.*

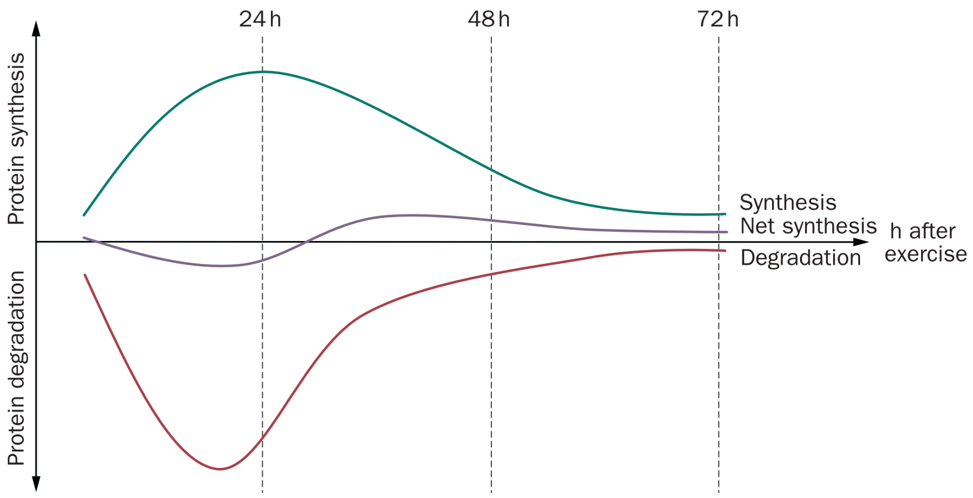

Figure 1. Schematic presentation of net balance between synthesis and degradation of collagen in response to loading (Magnusson et a. 2010[30])

Implication of increased restitution time and consequently reduced treatment frequency

Alteration of the restitution time in an exercise-based rehabilitation regime will naturally result in altered treatment frequency (times/week). Previous studies[19,32] reported that heavy slow resistance exercise 3 times per week was superior to daily eccentric exercises in tendinopathy patient's treatment satisfaction. The increased restitution length between sessions (three times pr week in contrast to training seven days a week) may have been a contributing factor to the improved outcomes, which would support the notion of reducing the number of training session. Fewer treatment sessions would also make the intervention less time consuming, requiring less effort and thereby making it more feasible for patients to combine with work and everyday life, potentially resulting in a greater adherence to the treatment.

A caveat to reducing the frequency of controlled exercises is that it leaves more time for other uncontrolled exercise activities. This could negate the effects of restitution and potentially exacerbate the tendinopathy. *Therefore, to ensure sufficiently tendon recovery between training session patients in the present study will have complete cessation of impact activity with high tendon loading (jumping, running) during the first 12 weeks of the intervention.*

Extended treatment duration and corticosteroid injection

A treatment duration of 8-12 weeks is typically applied in studies that investigate the effect of loading based treatment of tendinopathy[33]. Even though significant improvements are observed within 12 weeks of loading-based treatments, findings from previous studies in our lab on patellar tendinopathy[9,19,34] showed that some patients do not recovery after 12 weeks. Furthermore, a systematic review concluded that a duration >12 weeks of loading intervention might be more effective at inducing tissue alterations in healthy tendon[33], but this has never been investigated in persons with tendinopathy. *The present study will therefore include instructions to progress with loading-based rehabilitation beyond the 12 week intervention for patients responding to loading-based treatment that have not fully recovered after 12 week.*

Furthermore, previous studies in our lab on patellar tendinopathy[9,19,34] have shown that some patients do not respond to loading-based treatment of tendinopathy alone. Indeed, a recent study from the department[35] have shown that a combination of corticosteroid injections and controlled training proved superior to training or corticosteroid separately, in the treatment of plantar fasciitis. This approach may also be advantageous for patients with patellar tendinopathy not responding to loading-based treatment alone. *The present study will therefore offer patients who after 12 weeks did not respond to loading-based treatment alone to receive an add on treatment of corticosteroid injection in combination with continued loading-based treatment.*

4.0 Study Aim, Hypothesis and Objectives

4.1 Aim

The purpose of the present project is to investigate if the restitution time from loading in an exercise-based 12 weeks rehabilitation regime for patellar tendinopathy influences the clinical outcome, tendon structure and function.

4.2 Hypotheses

We hypothesize that greater restitution from loading (1 exercise day per week) will yield a greater positive clinical outcome, and tissue structure and function in patients with patellar tendinopathy compared to less restitution (3 exercise days per week), when impact activities are restricted in both groups.

4.3 Objectives

The *primary* objective is:

To assess if greater restitution from loading (1 exercise day per week) vs. less restitution (3 exercise days per week) during exercise-based treatment will yield a greater change in Victorian

Institute of Sports Assessment –patella (VISA-P) score from baseline to 12 weeks, in patients with chronic (symptoms > 3 month) patellar tendinopathy.

The *secondary* objectives are:
To asses if greater restitution from loading (1 exercise day per week) vs. less restitution (3 exercise days per week) during exercise-based treatment will yield a greater change on the following outcomes:

- Change in patient-evaluated symptoms, physical function, sports participation, and patient-evaluated improvement and treatment satisfaction
- Change in muscle and tendon function
  Change in patellar tendon structure and vascularization.

Other objectives (exploratory) of this study include:

- Investigate the feasibility of using Magnetic resonance imaging (MRI) with Blood Oxygenation Level Dependent (BOLD) imaging technique for mapping of brain structure, function and metabolism changes in chronic tendinopathy patients and for assessment of possible differences between patients that respond and those that do not respond to loading-based treatment.
- Test the feasibility and response to individualized treatment protocols focused on extended duration of the rehabilitation program combined with activity modification and load management after the 12-week intervention.
- Investigate the effect of add on treatment with corticosteroid (injection and 4 weeks of continued training) in a cohort of patients not responding to the initial 12-weeks loading-based induced treatment.

5.0 Study design

5.1 Trial design

This study is designed as a prospective, randomized, controlled, open label, superiority trial with a two-group parallel design and primary endpoint after 12 weeks. The study has two phases; The first phase includes the main trial in which a 12-week intervention period will be undertaken to test the hypotheses in patients with chronic patellar tendinopathy (symptoms > 3 months). The measurements (*see 8.0 Description of outcome*) will be obtained 3-4 days before and after the intervention period. Randomization will be performed as block randomization with a 1:1 allocation. At 12 weeks, a smaller group of patients (5 responding and 5 not-responding to the 12-week intervention period) will be offered to participate in sub-study 1. In this explorative cross-sectional study, the feasibility of mapping brain structure, function and metabolism using MRI BOLD imaging technique in chronic patellar tendinopathy patients will be assessed.

The second phase in the main study includes the follow-up from 12 week to the secondary endpoint at 52 weeks after baseline. During this period the participants will be monitored via questionnaires for treatment satisfaction and improvements at 4-week intervals. What treatment and the duration of treatment patients will receive in this phase is based on the concept of personalized medicine. We expect the majority of patient to continue in the group with loading-based intervention focusing on; education in load management (Stage 1), maintenance of exercise therapy and pain guided progression of impact loading (Stage 2), and return to sport (Stage 3). Progression is individualized based on patients own assessment of symptoms and will therefore vary in duration. A smaller group of the 52 patients from the main study is expected to be included in sub-study two.

Sub study two is designed as an observational cohort study. In this sub study, patients reporting no self-evaluated improvement after 12 weeks of loading-based treatment will be asked to be part of a group receiving corticosteroid injection treatment followed by continued exercise-based treatment and avoidance of impact loading. The cohort will further include patients that after week 20, 24, 28, 32, 36 and 40 respond that they have not achieved their Patient Acceptable Symptom State (PASS), and who wish to receive a corticosteroid injection. At the end of the corticosteroid-related treatment, patients will be offered the standard phase two intervention and we will continue to be monitored every 4 weeks until the same secondary endpoint as in the main study.

The overall trial design is illustrated in *figure 2*.

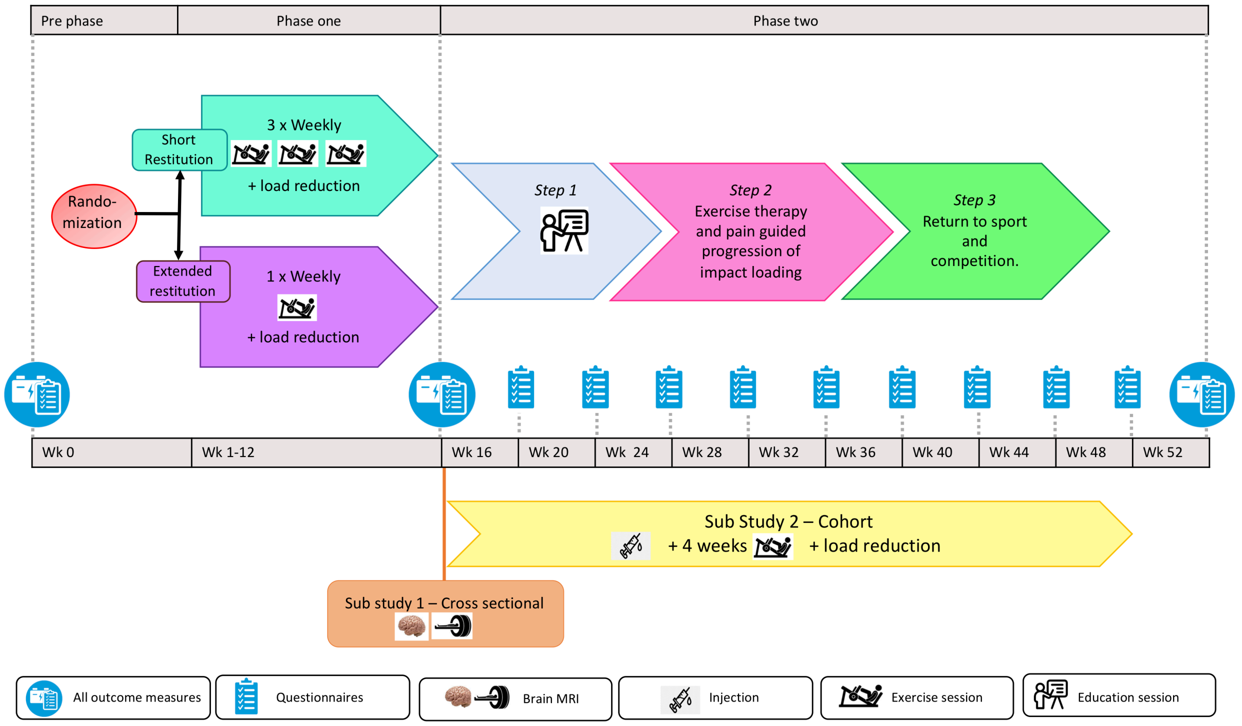

Figure 2: Illustration of trail design

5.2 Study setting

All the required methodology is available at Institute of Sports Medicine Copenhagen and Department of Physical and Occupational Therapy, Bispebjerg Frederiksberg Hospital. Based on previous work at the department[36] data collection is expected to take two years, and is planned to begin winter 2023.

5.3 Patient involvement

Patient partners were involved in the planning phase of this study to capture the patient perspective on the idea and purpose of the study, whether the intervention is feasible and the time requirement of participating in the study. The patient partners participated voluntarily in the process and the collaboration between patients and professionals in the project follows the European League Against Rheumatism recommendations (EULAR)[37] for the inclusion of patient representatives in scientific projects.

5.4 Study design considerations

Because the patients are not blinded in this superiority trial, we believe that patients that go into the short restitution group, might see this as a less effective treatment. Therefore, to reduce bias, the persons carrying out the measurements and the physiotherapists in charge of the intervention will be instructed to tell patients that both intervention programs are expected to provide beneficial results.

Further, in our experience patients may consider the corticoid steroid injection treatment as a more “effective” and requiring “less effort” than the loading-based rehabilitation, which could lead to purposely lowering satisfaction scores in order to receive the corticosteroid treatment. Therefore, we will only introduce sub-study two to the patients that are not improved at week 12 follow-up or not achieved a self-evaluated satisfactory result at the monthly follow-up from week 20 to 40. Therefore, information about sub-study two is available as a supplement to the main study “deltagerinformation” and will be given only to the eligible patients based on their rating (*see 6.1 Eligibility criteria*).

Finally, a contextual problem in the study is that a caveat to reducing the frequency of controlled exercises from 3 to 1 exercise day per week is that it leaves more time for other uncontrolled exercise activities. This could influence the pain level and negate the effects of restitution and potentially exacerbate the tendinopathy. Hence, to ensure sufficiently tendon recovery between training session patients in the present study will have complete cessation of impact activity with high tendon loading (jumping, running) during the first 12 weeks of the intervention, which might be challenging in these sport active patients. To mitigate this issue we will clearly explain how impact activities influence the treatment effect for the patients and be transparently how this influence the study results. Furthermore, patients will be provided with a program of exercises

that do not expose the tendon to impact activity, which they can perform if they wish to exercise beyond the controlled rehabilitation regime.

6.0 Participants

6.1 Eligibility criteria

A persons eligibility for study participation will be based on the following criteria:

<u>Inclusion criteria:</u>

- Sports active men and women.
- Age (18-60) years old.
- BMI (18.5-28)
- Understand and read Danish
- Uni- or bilateral patellar tendinopathy
- Symptom onset >90 days ago (defined as chronic[38])

<u>Exclusion criteria:</u>

- Patellar tendinopathy longer than 24 months
- Smoking
- Previous surgery in the knee on the ipsilateral side.
- Corticosteroid injection in the patellar tendon on the ipsilateral side within the last 6 months.
- Any confounding diagnosis to the knee joint
- Known arthritis
- Known diabetes
- Inability to follow rehabilitation or complete follow-ups
- Enrolled in a resistance based-rehabilitation program for the affected patellar tendon within the previous three month
- Have a work were it is not feasible to avoid pain provoking tasks

<u>Extra Inclusion and exclusion criteria sub-study one:</u>

Five patients from the main study, rating “improved” and “not improved” respectively, will be invited to participate in sub-study one.

Participants are dichotomized as improved if they rate themselves as ‘much improved’, ‘improved’ or ‘slightly improved’ (categories 5, 6 and 7) and categorized as not improved if they rate themselves from ‘unchanged’ to ‘much worse’ (categories 1 to 4) on the Likert scale.

Exclusion criteria: claustrophobia and pregnancy/breastfeeding.

<u>Inclusion criteria sub-study two:</u>
Patients from the main study, that rate themselves 'not improved' (dichotomized as described in sub-study one) in symptoms after 12 week and patients who have not achieved Patient Acceptable Symptom State (PASS) after week 20, 24, 28, 32, 36 and 40, will be invited to participate in sub-study two.
Exclusion criteria: pregnancy/breastfeeding or previously had an allergic reaction for steroid (Depomedrol) or local anesthesia treatment.

6.2 Diagnosis under study

Potential participants will be screened for the above-mentioned criteria during a phone screening, and at the pre-examination test day.

An experienced physician or sports physiotherapist will confirm the diagnosis based on predefined criteria;

- Existence of clinical signs of patellar tendinopathy:
  - Activity related pain in the patellar tendon.
  - Patients complaining of pain on palpation of the patellar tendon.

- At least one of the following three changes on ultrasound investigation.
  - Thickening of the AP diameter of the symptomatic area compared with the mid tendon level.
  - Presence of Power Doppler signal in the tendon on the symptomatic side.
  - Hypoechogenic area corresponding to the symptomatic area of the tendon.

6.3 Selection of study tendon

At inclusion a study patellar tendon will be selected and will be defined as the patient reported symptomatic patellar tendon. If the patient has bilateral symptoms, the most symptomatic tendon selected by the patient will be defined as study tendon. If both tendons are equivalent symptomatic the tendon with the most severe thickening of the tendon compared with the mid-tendon level will be defined as study tendon.

6.4 Allocation of participants and sequence generation

After baseline assessments, participants will be randomized to one of the two intervention groups:

A) Short restitution group (SR). This constitutes the currently accepted rehabilitation program of patellar tendinopathy with resistance training, three training session per week and is therefore considered the control group.

B) Extended restitution group (ER): Greater restitution from loading (1 exercise day per week)

Randomization procedure is performed using a computer-generated block randomization (block size is randomized to either 4 or 6) procedure. The allocation ratio will be 1:1 and stratified for the following baseline factors:

- Sex (male, female)
- Symptom duration (3-8, 9-24 months)

Senior researcher Rene B. Svensson will develop the randomization scheme for allocation of participants to the two groups in REDCap and will not be involved in the screening and inclusion process.

6.5 Blinding

It is not possible to blind an exercise intervention and therefore this trial will be carried out as an "open-label" trial where neither the patients, nor the physiotherapists providing the intervention will be blinded to treatment allocation. All patient-reported outcomes will be obtained electronically and blinded for members of the research team using REDCap. Furthermore, outcome assessors will be blinded to treatment allocation where possible and patients are requested not to disclose their allocation when outcomes are assessed. In addition, all baseline measurements will be collected before treatment allocation, and all data analyzed blinded. To test the blinding efficacy, the outcome assessors are asked what treatment strategy they think a patient has received after assessments.

6.6 Sample size

The main study is powered based on previous data, a within-subject standard deviation of the primary outcome (VISA-P) of 12.8 after 12 weeks is expected[9]. A sample size analysis reveals that each group should contain n=18 to detect a 13 points difference[39] (minimal clinically important difference) on VISA-P score[3] with an alpha level of 0.05 and a power/beta level of 0.80. To account for a 20% dropout rate and an estimated compliance rate of 75% (percentage of participants completing >80% of intervention) based on previous data[9], a total of 26 participants will be recruited for each group to ensure sufficient numbers for both intention-to-treat and per-protocol analysis.

Sub-study 1 is an explorative cross-sectional study investigate the feasibility of mapping brain structure. Therefore, number of participants is based on feasibility and will include 5 patients responding and 5 not-responding to the 12-week intervention period in the main study.

Participation in sub-study 1 will not influence how the patients progress in phase two of the main study or if they be enrolled in sub-study 2.

Number of participants included in sub-study 2 will be based on how many patients from the main study, that rate themselves 'not improved' (dichotomized as described in sub-study one) in symptoms after 12 week and how many patients who have not achieved Patient Acceptable Symptom State (PASS) after week 20, 24, 28, 32, 36 and 40, and agree to participate. Based on data from a previous study[36] this is expected to be approximately 20% corresponding 11 patients from main study.

6.7 Participants recruitment

The participant recruitment will be achieved in-house at Bispebjerg Frederiksberg Hospital from the sports medicine clinic, general practitioners, sports clubs, and online.

7.0 Study intervention

Intervention during the first phase of the study include the experimental exercise therapy and load reduction. The second phase include patient education in load management via the pain monitoring model, continued exercise therapy, gradually progressive activities (the activity ladder) and return to sport. The progression during the main track of the second phase consist of three steps and the pain monitoring model guide the patient through the steps. In addition, a description of the intervention in sub-study two is included in the description of the second phase.

7.1 Phase one (Main trial) - Exercise therapy with different restitution and load reduction

Exercise Therapy

The exercise program described below will be identical for both groups apart from the SR-group performing the resistance exercise three times per week and the ER-group performing an identical training regime but only once per week (increased restitution time). For the SR-group there must be at least 48 hours rest and for the ER-group 96 hours between each training session.

The intervention program will last for 12 weeks, and each session will consist of two exercises, *figure 3*

- One bilateral exercise; Leg press performed from 90° of knee flexion to 10° of knee fleksion and back again.
- One unilateral exercise (performed with both leg); Knee extension performed from 10° of knee flexion to 100° of knee flexion and back again

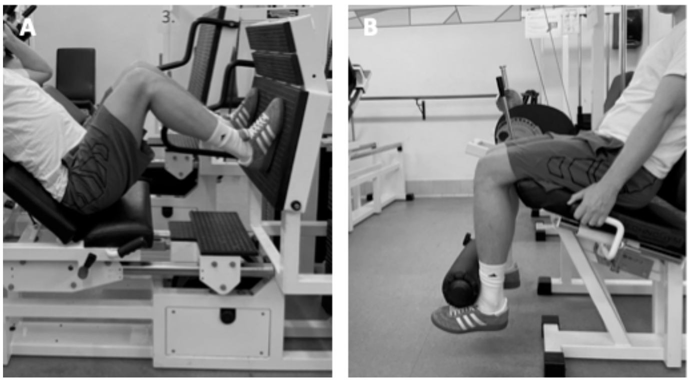

Figure 3: Depiction of applied exercises. A: Leg press, performed bilaterally; B: Knee extension performed unilaterally.

All patients will be instructed to spend three seconds completing each of the eccentric and concentric phases, respectively (i.e 6 s/repetition).
Patients in both groups begin with a 5-minute warm-up on a stationary bike ergometer (light-to-moderate intensity) followed by the three or four sets in each exercise with a 1-2 min rest between sets.

All training sessions, except those supervised, are performed in a commercial fitness center.

The loading program will be started at 60% of 1 RM and progressed to 75% of 1 RM during the first 3 weeks and maintained throughout the intervention period. The exact trainings load for each exercise is estimated based on the repetition maximum (RM) listed in table 1. Each exercise is initiated with loading that achieves muscle fatigue within 15 repetition in each set. An increase in load is recommended when the participants is able to perform 2 repetitions more than the desired number (i.e. 17 or more) with satisfying quality and within the limits of pain.

**Exercise protocol**				
**Week**	**1**	**2-3**	**4-5**	**6-12**
**Sets and reps**	3 x15	3 x 12	3x 10	4 x 10
**~ % of 1 RM**	60	70	75	75

Table 1: Loading protocol, identical for the two groups

Load reduction
Patients are not allowed to performed impact activities (running and jumping activities) that may provoke their patellar tendon outside of treatment. Likewise, patients will be guided in how to avoid strength training involving the quadriceps muscle. However, they will be encouraged and guided to perform non-impact activities (e.g. biking, swimming, rowing, strength training not involving the quadriceps muscle and any part of their normal training not including impact).

Maximum pain of 2 on numeric rating scale during, after, or the day after non-impact activities are accepted.

Tailoring
The load in each exercise will be tailored to the individual patient based upon individual strength and the load progression will be individually adjusted based on the load progression in *table 1*. The patients will be instructed that the load magnitude has to be high enough that they are only just able to perform the established repetitions pr set. If more repetitions can be performed, the load is increased in subsequent sets.

Further, load will be adjusted due to patient self-reported pain. Pain during treatment exercises is accepted to reach 5 on the NRS but pain and discomfort should not increase following cessation of training and if any training-induced pain did not subside 3 to 4 hours after the session, the load will be reduced during the next session.

Adherence
To increase adherence, specifically in relation to how the exercises are performed, that the prescribed loads are achieved and that participants refrain from impact activities, one session in weeks 1, 3 and 6 will be carried out under supervision of a physiotherapist one patient at a time, and delivered face to face. In week 2 and 4 the patient will be remined of changing the training load by a text message and in week 9 the physiotherapy will follow-up on the training intervention, compliance and to what extent complied with the load-reduction with a phone-call. Moreover, compliance with exercise and activity modification will be tracked using a training diary. Patients will be asked to record the number and load of the treatment exercises completed and whether they performed running, jumping or other activities outside of the intervention. In addition, pain level is rated prior to, during and after their activities.
The patient will be defined as compliant with the treatment if they overall have performed at least 80% of the prescribed exercise sessions. I addition, patients will only be defined as 'compliant' if they complied with with the load-reduction for at least 10 of the 12 weeks.

7.2 Phase 2 – Follow-up treatment in the main study

Stage 1 - Education component - pain-guided activity modification
A physiotherapist will educate the patient in the pain monitoring model (Figure 4) and introduce how this tool can help them to progress and regress activities and interpret symptoms.

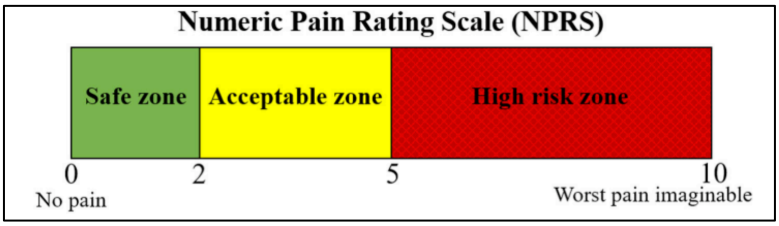

Figure 4: Scale used as pain monitoring tool (modified from Thomee et al 1997[40])

The physiotherapist will explain why it is important to start back slowly and a progression plan gradually exposing the patient to activities with higher patella tendon loads (The activity ladder) will be introduced, *Figure 5*

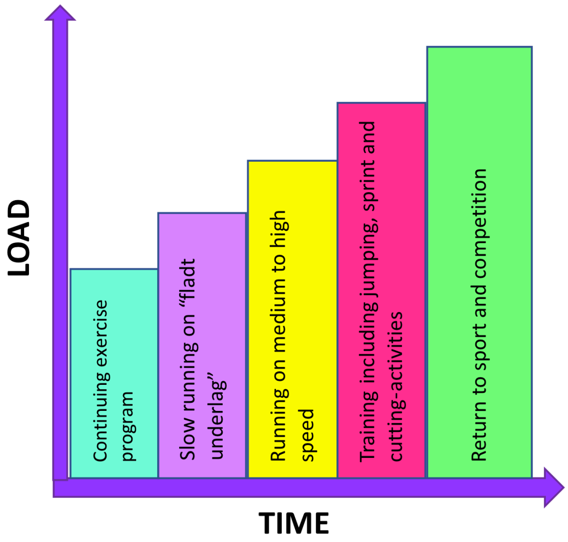

Figure 5 : Activity ladder

Moving to next step on the activity ladder will be based on the patient's own assessment of symptoms (pain and morning stiffness). If they can perform an activity within the "Safe Zone" on the pain monitoring model, without a pain flare-up or increased tendon stiffness they can progress to next level.

Stage 2 - Maintenance of Exercise therapy and pain guided progression of impact loading
During step two, patients perform exercise therapy and pain guided progression of impact loading on their own. The pain monitoring tool guides the patient through the 4 steps in the activity ladder. When patients are able to perform training including jumping, sprint and cutting-activities they can progress to *Step 3 - Return to sport and competition*.

Stage 3 - Return to sport and competition
Patients can gradually participate in sport specific training if the activities can be performed with no pain, or a minimal pain within the "safe zone" during, immediately after and the morning after activities.

They are recommended to slowly increase their training as follow:

- Taking part in the warm-up and approximately 15 minutes of the training.
- Increase the amount of training by around 5 minutes pr session/week? If no worsening of the tendon pain.

- When they are able to participate in a full training without knee pain for two weeks in a row, they can start return to match or competition. They are recommended also to start this part slowly by e.g. only participate in one half as a start.

7.3 Sub-study two – Add on treatment of injection, exercise therapy and load reduction

All patients will follow the SR exercise regime and load reduction as described in Phase one for minimum 4 weeks. The treatment is supplemented with ultrasound-guided injection of 1 ml. Lidokain + 1 ml (40 mg/ml) corticosteroid, in total 2 ml (standard treatment). A maximum of 3 injection will be offered every 4. week as described below.

Injection of 2 ml with corticosteroid

The injection consists of a mixture of the corticosteroid: Depo-Medrol 1 ml and 1 ml Lidokain 1%. The mixture will be injected below the tendon corresponding to the thickest place under ultrasound guidance. The first corticosteroid injection is given to all patients included in sub-study two. The second injection after 4 weeks is only provided if the tendon structure modifications are unaltered. The third injection is only provided if there in addition to continued unaltered tendon structure are pain (NRS >2) in the morning or during activity. Thus, a maximum of 3 corticosteroid injections are given corresponding to usual standard treatment at the department. The patients are not allowed any training/loading for the first 48 hours after an injection.

Injection procedure

The patient is placed in a supine position with a 90° flexion on the hip and knee joint. The Ultrasound transducer is placed longitudinally over the tendon so that the apex patella and the thickened area is visible in the scan plan. After 3 times disinfection of the skin the needle with 2ml local anesthetic and corticosteroid is inserted approximately 1 cm laterally for ligamentum patella and 0,5 cm distal for apex patella. The needle is placed just below the ligamentum patella at the attachment to patella so the needle tip ("a white dot") is visible in the center of the scan field. Correction of the needle placement takes place until the tip is visible just below the ligamentum patella after which the entire dose is injected slowly.

8.0 Outcome assessment variables

8.1 Primary Outcome

The primary outcome is assessed at week 12 as change from baseline in Victorian Institute of Sports Assessment –patella (VISA-P) total score.

8.2 Secondary outcome

The following outcome are assessed as secondary outcome:

- Change from baseline in VISA-P at week 16 and 52.
- Change from baseline in truncated VISA-P (questions 2-6) at week 12, 16 and 52.
- Change from baseline in pain rating on numeric rating scale (NRS) during preferred sport, rest and daily activities at week 12, 16, 20, 24, 28, 32, 36, 40, 44, 48, 52.
- Change in Self-reported improvement from baseline on a GROC scale at week 12, 16, 52.
- Number of sports participation hours per week (training and competition), type of sport and intensity at week 12, 16, 20, 24, 28, 32, 36, 40, 44, 48, 52.
- Patient Acceptable Symptom State (PASS) at week 12, 16, 20, 24, 28, 32, 36, 40, 44, 48, 52.
- Registration of care-seeking behavior and treatment received from last follow-up at week 12, 16, 20, 24, 28, 32, 36, 40, 44, 48, 52.
- Change from baseline in pain during SLDS test at week 12 and 52.
- Change from baseline in maximal muscle strength at week 12 and 52.
- Chang from baseline in tendon thickness and neovascularization at week 12 and 52.
- Change from baseline in microvascular blood flow
- Change from baseline in jump height at week 12 and 52.

8.3 Other outcomes

Only for patients participating in sub-study one:

- If it is feasible to map brain structure, function and metabolism changes in patient with chronic patellar tendinopathy using MRI.
- Assess if Patient Acceptable Symptom State (PASS) is correlated with brain structure, function and metabolism MRI findings.

Only for patients participating in sub-study two:

- Number of injections received from week 12-52
- Change from 1. injection in tendon thickness and neovascularization at week 4 and 8 after the injection.
- Change from 1 injection in self-evaluated PASS and improvement at week 4 and 8 after the injection.
- Registration of pain rating on numeric rating scale (NRS) during injection.

9.0 Description of the outcome

9.1 Clinical picture

Standard diagnose specific questionnaire; Victorian Institute of Sports Assessment –patella (VISA-P) will be used to asses symptoms, function and the ability to participate in sports[41].

VISA-P has shown psychometric issues (data not published), nevertheless, this questionnaire was chosen because it is the only diagnose specific questionnaire, it remains the standard PROM for this condition and previous data is available to allow for sample-size calculation. Because of the issues of the VISA-P, a truncated version (only including Item 2-6), which was confirmed more adequate in a recent analysis (data not published), will also be included in the present study.

Maximal tendon pain during preferred sport, rest, daily activities and treatment will be assessed on a 0 to 10 numeric rating scale (NRS).
Furthermore, sports participation in hours per week (training and competition), type of sport, intensity and care-seeking behavior and treatment received between follow-ups will be registered.

The Global Rating of change (GROC) will be used to measure patient self-reported improvement on a 7-point Likert scale ranging from 'much improved' to 'much worse'. Patients will be dichotomized as improved if they rate themselves as 'much improved', 'improved' or 'slightly improved' (categories 5, 6 and 7) and categorized as not improved if they rate themselves from *'unchanged'* to 'much worse' (categories 1 to 4).

Self-evaluated satisfaction with treatment result will be evaluated by Patient Acceptable Symptom State (PASS)[42] on a 5-point Likert scale ranging from 'very satisfied' to 'very unsaticfied'. Patient will be dichotomized as satisfied if they rate themselves as 'very satisfies', 'satisfied' or 'neutral' or as unsatisfied id the rate themselves 'unsatisfied' or 'very unsatisfied'. PASS score will be used as a measure of if patients evaluate the treatment to be a satisfactory result and feel no need for further treatment.

9.2 Functional test

A reliable patellar tendon pain provocation test, the single-leg decline squat (SLDS)[43], will be used to assess pain during function. Furthermore, maximal muscle strength is obtained during standardized maximal voluntary contractions[44].
Furthermore, a Counter movement Jump test will be used to assess patellar tendinopathy caused functional deficits[45].

9.3 Imaging technologies

Ultrasonography

Grey scale for tendon thickness and power Doppler for neovascularization will be obtained as previously described[9]. In addition B-Flow measurement will be used to assess microvascularisation of the patellar tendon.

Magnetic resonance imaging (Only patients included in sub-study one)
Magnetic resonance imaging with Blood Oxygenation Level Dependent (BOLD) imaging technique will be used for mapping brain structure, function and metabolism[25].

10.0 Study procedure

10.1 First contact

Patients, who express interest in participating in this research project, will firstly go through a phone screening. During this screening they will be informed about the project and the inclusion criteria and they will have the written information sent ("Deltagerinformation") and will be given about a week to consider their further interest. If they are still interested in participating, they will be invited to a briefing/prescreening, to which they are informed that they are allowed to bring an attendant.

10.2 Assessment schedule

Participants assessment schedule will be performed as in the schematic diagram *Figure 6*.

Briefing
The briefing will take place at the Institute of Sports Medicine, Bispebjerg Hospital. The information will be given in an uninterrupted setting in a private room by the primary investigator. On the briefing day the participant will receive oral information about the project and be interviewed to ensure that they match the inclusion criteria. If the participant wishes to participate in the study, they have to sign a declaration of consent ("Samtykkeerklæring"). The participants will be informed of their right for time (minimum 24 hours) to consider participating for the study and no tests will be performed, before the declaration of consent is signed. Furthermore, the participants will be informed that they are always able to contact the primary investigator for further information, and that they can at any time leave the experiment without further explanation and without any impact on future investigations and/or treatments.

Pre-exam
A pre-exam at the Institute of Sports Medicine, Bispebjerg Hospital, will fulfill the screening of the patients and include confirmation of the diagnoses (*ad. 6.2 Diagnosis under study*). If the patient does not fulfill the inclusion criteria after the examination, the patient will be excluded from the study. Furthermore, the screening will include registration of sex, age, height, weight, previous and/or systemic disease, tobacco, medicine, questionnaire (previous injuries and disease history, training history, training type and frequency), questionnaire (physical activity level) and registration of pain level on a numeric rating scale (NRS) in rest and during activity. Lastly, participants will be informed to avoid strenuous physical activity 24 hours before the experiment day.

Experiment day 1 - baseline (3-4 days before the intervention period)
Patients will meet at the Institute of Sports Medicine, Bispebjerg Hospital. First, patients will complete written VISA-P, NRS scale, patients self-evaluated satisfaction (PASS) and registration of activity level of sporting activities electronically via REDCap with no investigator assistance. Thereafter, ultrasonography scan, muscle strength and functional tests will be examined.
Intervention period
See section "*7.0 Study intervention"* for details

Experiment day 2 - primary endpoint (3-4 days after the intervention period)
Experiment day two will proceed exactly as experiment day one. Further, the patients will score self-reported improvement perception and care-seeking behavior and treatment received during the time between last and current follow-up as a part of the questionnaire.

For patients included in *Sub-study one* an additional MRI scan will be scheduled on a sperate day within the following week. The MRI scan will be performed at the department of radiology Bispebjerg Frederiksberg Hospital. Patients will be informed to avoid strenuous physical activity 24 hours before they meet and furthermore be seated in the waiting room 15 minutes before the assessment.

Extra experiment days for patient included in sub-study two (between week 12 and 40)
Patients who have not improve at week 12 follow-up or not achieved satisfactory results on assessment at week 20 to 40 are offered to continue in sub study two. Patients will receive oral information about the sub-study by the primary investigator and they will have the written information ("Tillæg til skriftlig deltagerinformation; Delstudie 2, Injektion som supplement til aflastning og træningsbaseret behandling ").
If the patient wishes to continue in sub-study two:

- They have to sign a declaration of consent ("Samtykkeerklæring delstudie 2"). The participants will be informed of their right for time (minimum 24 hours) to consider participating in the study and no examination will be performed, before the declaration of consent is signed.
- Patients who want to receive an ultrasound-guided corticosteroid injection will be examined by the responsible clinical investigator, who is a trained rheumatologist at the department. The clinical investigator will also explain the treatment (ultrasound-guided injection, load reduction and continued exercises) and the risks.
- The patients who agree to treatment must do a new baseline examination, where the patella tendon is ultrasound scanned by the doctor and tendon thickness and power Doppler are determined.

- The patients receives the intervention as described in "7.3 Sub-study two – Add on treatment of injection, exercise therapy and load reduction"
- Patients are checked by the doctor after 4 and 8 weeks. The ultrasound scan and the PASS is repeated at each visit. Injections will be repeated as needed at the 4 and 8 weeks follow-up.

Post-intervention monitoring week 16, 20, 24, 28, 32, 36, 40, 44, 48
NRS-score, patient self-reported improvement (GROC), patients self-evaluated satisfaction (PASS) and registration of physical activity level and care-seeking behavior and treatment received during the time between last and current follow-up will be sent to the patients electronically via RedCAP and will be completed with no investigators assistance.

Follow-up - secondary endpoint (52 weeks after baseline)
Follow-up 52 week after baseline will proceed exactly as experiment day two and include all participants regardless of their participation in sub-studies.

10.3 Participants time requirements

For the individual participant, the timespan of the project is approximately twelve months, and include nine visits at Bispebjerg Frederiksberg Hospital and a 12 week long rehabilitation program with one or three training session pr. week.

For all patient included in the study:

- First contact, briefing and pre-exam: Approximately 2 hours.
- Experiment days 1, 2 and 52 week follow-up: Approximately 3 hours each.
- Phase 1 intervention program week 1-12: Each training session takes approximately 45-60 minutes. 3 of the sessions will be superviced at Bispebjerg hospital.
- Follow-up (mailed written questionnaires) every fourth week (week 16-48): Approximately 15 minutes each.
- Phase 2 - Education by a physiotherapist in individualized treatment: Approximately 30-45 minutes 20

For patients included in sub study-one will be added on extra visit at experiment day 2:

- One MRI examination: Approximately 60 minutes

For patients included in sub-study two will be added 3 visits:

- 3 Examination/injection with a rheumatologist: Approximately 20 minutes each.
- Extended intervention program 4 weeks: Each training session take approximately 45-60 minutes.

10.5 Treatment visit window

- The exercise sessions is optimally carried out at fixed week days +/- 1 day and should be carried out with a minimum of 48 (SR-group) or 96 (ER-group) restitution between training session.
- The education session starting up phase 2 intervention will be scheduled within a week after Experiment day 2.

		STUDY PERIOD													
	Enrolment	Allocation	Post-allocation												Close-out
**TIMEPOINT**	*February 2023 - February 2025*	*February 2023 - February 2025*	*0 wk*	*12 wk*	*16 wk*	*20 wk*	*24 wk*	*28 wk*	*32 wk*		*36 wk*	*40 wk*	*44 wk*	*48 wk*	*February 2024- February 2026 (52-week follow-up)*
			Phase 1 inter-vention 12 wk		*Phase 2 Individualized Intervention between wk 12 and 52 + intervention sub-study 2*										
**ENROLMENT**															
Eligibility screen	X														
Informed consent	X														
Allocation		X													
**INTERVENTIONS**															
High Rest Group															
Low Rest Group															
Individualized															
Injection add on															
**ASSESSMENT** (Main Study)															
Diagnosis	X														
Questionaries			X	X	X	X	X	X	X	X	X	X	X	X	X
Functional test			X	X											X
Imaging			X	X											X
Pain pressure threshold			X	X											X
**ASSESSMENT** (Sub-study one)															
MRI brain				X											

**ASSESSMENT** (Sub-study two)															
Ultrasound*															
PASS*															

Figure 6. Schedule of enrolment, intervention and assessment. *outcome assessment will vary within the time span, depending of inclusion timepoint in sub-study two.

10.4 Assessment window

The visit windows are as follows:

- Pre-exam will be done no more than 4 weeks before randomization.
- Baseline assessment will be taken no more than 7 days before intervention.
- The 12-week assessment will be taken no more than 3-4 days after intervention.
- The monthly follow-up (week 16- 48) can be taken up to 7 days after the scheduled date.
- 52-week follow-up can be taken within +/- 14 days of the scheduled date.

10.5 Treatment visit window

- The exercise sessions is optimally carried out at fixed week days +/- 1 day and should be carried out with a minimum of 48 (SR-group) or 96 (ER-group) restitution between training session.
- The education session starting up phase 2 intervention will be scheduled within a week after Experiment day 2.

10.6 Time plan for recruiting patients and testing

Data collection is expected to begin in the winter of 2023 and run through the winter of 2025.

10.7 Logistics

All the required methodology is available at Institute of Sports Medicine Copenhagen, Department of Physical and Occupational Therapy and Orthopedic Department, Bispebjerg and Frederiksberg Hospital.

11.0 Discontinuation

11.1 Participant withdrawal

A participant may withdraw from the study at any time without any impacting on any future investigations and/or treatments at the site, by the Investigators in this study or by other staff associated with the study. If a participant withdraws from the study, the procedures outlined for the closest assessment visit is sought to be completed within 2 weeks, and preferably prior to the initiation of another therapy. However, these procedures should not interfere with the initiation of any new treatments or therapeutic modalities that the investigator feels are necessary to treat the participant's condition. It is important to avoid any lost to follow-up participants for meaningful analysis of the study.

11.2 Individual participant discontinuation

The investigator may discontinue any participant's participation for any reason, including an adverse event (AE), safety concerns or failure to comply with the protocol.
Participants will be discontinued from the study immediately if any of the following occur:

- Clinically significant abnormal results or AEs, which rule out continuation of the study treatment, as determined by the investigator.
- Other illness
- Failure to adhere to the protocol.

If at any point in time between randomization and the week-12 visit the investigator feels that the patient's clinical course is not acceptable within the normally applied paradigms of Patellar tendinopathy, the patient should be taken out of the study. The clinician's judgment will be required to decide on a case-by-case basis whether to implement this step or not. It is important to avoid any loss to follow-up participants for meaningful analysis of the study.

Discontinued participants can be replaced by new participants. The reason for an eventual discontinuation is registered.

11.3 Discontinuation of Entire Study

The primary investigator has the right to terminate this study at any time. Reasons may include the following, but are not restricted to:

- The incidence of events in this or other studies that indicate a potential health hazard to participants.
- Unsatisfactory participant enrolment.

12.0 Statistical methods

Appropriate statistics to evaluate clinical change with treatment over timer will be applied. Analyses will be performed both on intention-to-treat and per-protocol (participants completing

>80% of intervention) basis. The primary outcome will be assessed on the intention-to-treat basis on the interaction term of a 2-way (group, time) mixed model of VISA-P at baseline and 12 weeks.

13.0 Ethical aspects, risks and side effects

13.1 Research ethic intervention and assessment

Firstly it should be pointed out, that by enrolment in any research project, one can be subjected to unwanted risks or side effects.

Non-invasive measurements

Questionnaires and ultrasound assessment : These measurements are all non-invasive and not associated with any known risks.

Training and muscle strength assessment

For patients in both training groups, the resistance training may produce light muscle soreness in the days after training. The soreness is harmless and will decrease during the intervention period. The assessment of maximum voluntary contraction strength, rate of force development and single leg decline squad testing may also induce similar temporary soreness as the resistance training.

Injection

For patients included in sub-study two there will be a risk of discomfort and pain after the injection. However, the discomfort usually disappear after 1-2 days weeks after the injection. Every time an injection is given, a risk of infection exist. However, the risk is estimated to be less than 1:25.000 injections and can be treated with antibiotics as needed.

To ensure patient safety, contraindications; wounds in the area, infection in the area, insulin-requiring diabetes and AK treatment will be checked before injection. All adverse reactions to the injections will be recorded.

Training and injection

Likewise for patients included in sub-study two there is a risk that reduced tendinopathic pain following the injection of corticosteroid, causes patients to progress training too quickly, which can result in poor long-term outcomes and in very rare cases tendon rupture. However, this is avoided by a carefully supervised training progression and cessation of impact loading (running and jumping), which ensures that there is no increased risk of tendon rupture.

Magnetic Resonance imaging:

Patients included in sub study one will undergo one MRI scanning in continuation of the second experiment day. The patient will be placed in a magnetic field, which normally cannot be perceived. Individuals with metal pieces on their bodies e.g. crews/ plates due to surgery would be able to feel the magnetic field and can therefore not be included into the study. We will question

the patients about the possibility of metal pieces in their bodies prior to the scanning. The MRI scanning is not associated with any known risks but is not comfortable for individual suffering from claustrophobia. Because, we see a picture of the anatomical structure of the brain, we can in rare cases find some abnormal changes that were not known in advance. Only extremely rarely will these be thought to have influence on general health. However, it is the practice of the MR Research Section to inform about such findings. Therefore, consent to participate in the trial automatically also consent to receive information about such findings and to the information then be sent to your own doctor and/ or relevant hospital ward (unless the patient refuses to do so while giving consent) which could potentially affect future opportunities to take out private health or life insurance. It is emphasized, however, that the MRI scan in connection with the experiment is not performed as an actual diagnostic scan and cannot be perceived as a health check.

13.2 Risks contra benefits of this study

The results from this project will reveal how restitution in a loading-based rehabilitation regime for patellar tendinopathy influences the clinical outcome, tendon structure and function. This will help establish the most efficient treatment recommendations.
On the individual level, patients participating in the project will benefit from a carefully designed rehabilitation program with regular supervision, which is beyond standard treatment. Normally, these patients would receive one instruction from a physiotherapist at the in-house sports clinic and would not receive close follow up and careful supervision over several weeks. Furthermore, patients participating in the study will have a careful clinical examination both at the start and at the end of the study and will be given feedback on the results.
Tendinopathy is a substantial clinical and socio-economic problem since it is a very common condition and the optimal treatment is still unknown. On the general population level, this study will give important information on the effect of various treatment options and the clinical outcome and tissue response to such treatments. This will be of importance of the physically active population, in which tendinopathy can limit exercise participation and ability to work for month and even years. It is expected that that the benefits will outweigh the risks and potential side effects associated with this study and the study is considered ethically justifiable.

14.0 Regulatory standards

14.1 Storage of data

Data will be coded and stored in a pseudonymized form, which can be identified by the primary investigator. Therefore, the data in this study is not depersonalized. When all participants data is analyzed, and the practical experiment is completed, data will be anonymized at latest 3 years after the last participant has completed the trial. The participants will be protected, according to

“Databeskyttelsesforordningen og Databeskyttelsesloven”. The project is also reported to the public data controller at Region H and permission will be obtained.

14.2 Patient insurance

All participants are covered by the Patient Compensation Act (Patienterstatningen).

14.3 Roles and responsibilities

The study will take place at the Institute of Sports Medicine Copenhagen and Department of Physical and Occupational Therapy, Bispebjerg Frederiksberg University Hospital. The initiative for this project was taken by Post doc. Anne-Sofie Agergaard and professor S. Peter Magnusson.
There is no connection between anyone in the scientific staff and the parties who funded the projects, just as neither of the parties who funded this project, financially or in any other way, will influence the results or data analysis.
The participants will not receive any financial benefits, or other compensation for participation in this study.

14.4 Funding

The total amount of this project is estimated to be 1,810.950 DKK. The general funding for salaries and running costs for the study is obtained from Lundbeckpuljen til sundhedsfaglig forskning og Rigshospitalet/UCSF (300.000 DDK), Bispebjerg Frederiksberg Hospitaler Frie midler (75.000 kr), and Department of Physical and Occupational Therapy, Bispebjerg Hospital (288.000 DKK).
Further funding will be applied. The ethical committee will be informed about obtained foundation grants, including the name of the foundation and the amount of money granted. In addition, the written information (deltagerinformation) will be updated following funding including information on the name of the foundation and the amount granted.

15.0 Publication of results

When the project has finished, all the participants who, through their declaration of consent, confirmed that they wanted to be informed about the study results will receive written information with results and overall conclusions from the study. The achieved results will be published in an anonymized form, just as the results presented in other professional contexts will be anonymized. We will publish all the results, positive, negative and inconclusive in international peer reviewed journals, as well as present the data at national and international scientific conferences and meetings. If the results are not accepted in an international peer reviewed

journal, although not expected, the data will be published at http://www.clinicaltrials.gov.

16.0 Completion of the study

The end-of-study is defined as the date of the last participant's last scheduled visit or the actual date of follow-up contact, whichever is longer.

17.0 References

1. Finni, T., Komi, P. V & Lepola, V. In vivo human triceps surae and quadriceps femoris muscle function in a squat jump and counter movement jump. *Eur. J. Appl. Physiol.* **83**, 416–26 (2000).
2. Khan, K. & Cook, J. The painful nonruptured tendon: clinical aspects. *Clin. Sports Med.* **22**, 711–25 (2003).
3. Scott, A. *et al.* Sports and exercise-related tendinopathies: A review of selected topical issues by participants of the second International Scientific Tendinopathy Symposium (ISTS) Vancouver 2012. *Br. J. Sports Med.* **47**, 536–544 (2013).
4. Riel, H., Lindstrøm, C. F., Rathleff, M. S., Jensen, M. B. & Olesen, J. L. Prevalence and incidence rate of lower-extremity tendinopathies in a Danish general practice: A registry-based study. *BMC Musculoskelet. Disord.* **20**, 4–9 (2019).
5. Kettunen, J. A., Kvist, M., Alanen, E. & Kujala, U. M. Long-term prognosis for jumper's knee in male athletes. A prospective follow-up study. *Am. J. Sports Med.* **30**, 689–92 (2002).
6. De Vries, A. J. *et al.* The impact of patellar tendinopathy on sports and work performance in active athletes. *Res. Sport. Med.* **25**, 253–265 (2017).
7. Hopkins, C. *et al.* Critical review on the socio-economic impact of tendinopathy. *Asia-Pacific J. Sport. Med. Arthrosc. Rehabil. Technol.* **4**, 9–20 (2016).
8. Malliaras, P., Barton, C. J., Reeves, N. D. & Langberg, H. Achilles and patellar tendinopathy loading programmes: A systematic review comparing clinical outcomes and identifying potential mechanisms for effectiveness. *Sport. Med.* **43**, 267–286 (2013).
9. Agergaard, A.-S. *et al.* Clinical Outcomes, Structure, and Function Improve With Both Heavy and Moderate Loads in the Treatment of Patellar Tendinopathy: A Randomized Clinical Trial. *Am. J. Sports Med.* **49**, 982–993 (2021).
10. Larsson, M. E. H., Käll, I. & Nilsson-Helander, K. Treatment of patellar tendinopathy-a systematic review of randomized controlled trials. *Knee Surgery, Sport. Traumatol. Arthrosc.* **20**, 1632–1646 (2012).
11. Gaida, J. E. & Cook, J. Treatment options for patellar tendinopathy: Critical review. *Curr. Sports Med. Rep.* **10**, 255–270 (2011).
12. Andriolo, L. *et al.* Nonsurgical Treatments of Patellar Tendinopathy: Multiple Injections of Platelet-Rich Plasma Are a Suitable Option: A Systematic Review and Meta-analysis. *Am. J. Sports Med.* **47**, 1001–1018 (2019).
13. Everhart, J. S. *et al.* Treatment Options for Patellar Tendinopathy: A Systematic Review. *Arthrosc. - J. Arthrosc. Relat. Surg.* **33**, 861–872 (2017).
14. Larsson, M. E. H., Käll, I. & Nilsson-Helander, K. Treatment of patellar tendinopathy--a systematic review of randomized controlled trials. *Knee Surg. Sports Traumatol. Arthrosc.* **20**, 1632–46 (2012).
15. Stanish, W. D., Rubinovich, R. M. & Curwin, S. Eccentric exercise in chronic tendinitis. *Clin. Orthop. Relat. Res.* 65–8 (1986). doi:10.1097/00003086-198607000-00014
16. Alfredson, H., Pietilä, T., Jonsson, P. & Lorentzon, R. Heavy-load eccentric calf muscle training for the treatment of chronic Achilles tendinosis. *Am. J. Sports Med.* **26**, 360–6 (1998).
17. Garma, T. *et al.* Similar acute molecular responses to equivalent volumes of isometric, lengthening, or shortening mode resistance exercise. *J. Appl. Physiol.* **102**, 135–43 (2007).
18. Heinemeier, K. M. *et al.* Expression of collagen and related growth factors in rat tendon and

skeletal muscle in response to specific contraction types. *J. Physiol.* **582**, 1303–1316 (2007).
19. Kongsgaard, M. *et al.* Corticosteroid injections, eccentric decline squat training and heavy slow resistance training in patellar tendinopathy. *Scand. J. Med. Sci. Sport.* **19**, 790–802 (2009).
20. Arampatzis, A., Karamanidis, K. & Albracht, K. Adaptational responses of the human Achilles tendon by modulation of the applied cyclic strain magnitude. *J. Exp. Biol.* **210**, 2743–2753 (2007).
21. Joshi, S. D. & Webb, K. Variation of cyclic strain parameters regulates development of elastic modulus in fibroblast/substrate constructs. *J. Orthop. Res.* **26**, 1105–13 (2008).
22. Wang, J. H.-C. Mechanobiology of tendon. *J. Biomech.* **39**, 1563–82 (2006).
23. Webb, K. *et al.* Cyclic strain increases fibroblast proliferation, matrix accumulation, and elastic modulus of fibroblast-seeded polyurethane constructs. *J. Biomech.* **39**, 1136–1144 (2006).
24. Spisak, T. *et al.* Pain-free resting-state functional brain connectivity predicts individual pain sensitivity. *Nat. Commun.* **11**, (2020).
25. Boraxbekk, C. J., Salami, A., Wåhlin, A. & Nyberg, L. Physical activity over a decade modifies age-related decline in perfusion, gray matter volume, and functional connectivity of the posterior default-mode network-A multimodal approach. *Neuroimage* **131**, 133–141 (2016).
26. Rhea, M. R., Alvar, B. A., Burkett, L. N. & Ball, S. D. A meta-analysis to determine the dose response for strength development. *Med. Sci. Sports Exerc.* **35**, 456–464 (2003).
27. Bojsen-Møller, J., Kalliokoski, K. K., Seppänen, M., Kjaer, M. & Magnusson, S. P. Low-intensity tensile loading increases intratendinous glucose uptake in the Achilles tendon. *J. Appl. Physiol.* **101**, 196–201 (2006).
28. Langberg, H., Skovgaard, D., Petersen, L. J., Bulow, J. & Kjaer, M. Type I collagen synthesis and degradation in peritendinous tissue after exercise determined by microdialysis in humans. *J. Physiol.* **521 Pt 1**, 299–306 (1999).
29. Miller, B. F. *et al.* Coordinated collagen and muscle protein synthesis in human patella tendon and quadriceps muscle after exercise. *J. Physiol.* **567**, 1021–33 (2005).
30. Magnusson, S. P., Langberg, H. & Kjaer, M. The pathogenesis of tendinopathy: Balancing the response to loading. *Nat. Rev. Rheumatol.* **6**, 262–268 (2010).
31. Silbernagel, K. G., Thomeé, R., Thomeé, P. & Karlsson, J. Eccentric overload training for patients with chronic Achilles tendon pain--a randomised controlled study with reliability testing of the evaluation methods. *Scand. J. Med. Sci. Sports* **11**, 197–206 (2001).
32. Beyer, R. *et al.* Heavy slow resistance versus eccentric training as treatment for achilles tendinopathy: A randomized controlled trial. *Am. J. Sports Med.* **43**, 1704–1711 (2015).
33. Bohm, S., Mersmann, F. & Arampatzis, A. Human tendon adaptation in response to mechanical loading: a systematic review and meta-analysis of exercise intervention studies on healthy adults. *Sport. Med. - Open* **1**, (2015).
34. Olesen, J. L. *et al.* No Treatment Benefits of Local Administration of Insulin-like Growth Factor-1 in Addition to Heavy Slow Resistance Training in Tendinopathic Human Patellar Tendons: A Randomized, Double-Blind, Placebo-Controlled Trial With 1-Year Follow-up. *Am. J. Sports Med.* **49**, 2361–2370 (2021).
35. Johannsen, F. E. *et al.* Corticosteroid injection is the best treatment in plantar fasciitis if combined with controlled training. *Knee Surgery, Sport. Traumatol. Arthrosc.* **27**, 5–12 (2019).

36. Agergaard, A. S. *et al.* Clinical Outcomes, Structure, and Function Improve With Both Heavy and Moderate Loads in the Treatment of Patellar Tendinopathy: A Randomized Clinical Trial. *Am. J. Sports Med.* **49**, 982–993 (2021).
37. De Wit, M. P. T. *et al.* European League Against Rheumatism recommendations for the inclusion of patient representatives in scientific projects. *Ann. Rheum. Dis.* **70**, 722–726 (2011).
38. Blazina, M. E., Kerlan, R. K., Jobe, F. W., Carter, V. S. & Carlson, G. J. Jumper's knee. *Orthop. Clin. North Am.* **4**, 665–78 (1973).
39. Hernandez-Sanchez, S., Hidalgo, M. D. & Gomez, A. Responsiveness of the VISA-P scale for patellar tendinopathy in athletes. *Br. J. Sports Med.* **48**, 453–457 (2014).
40. Thomeé, R. A comprehensive treatment approach for patellofemoral pain syndrome in young women. *Phys. Ther.* **77**, 1690–703 (1997).
41. Visentini, P. J. *et al.* The VISA score: an index of severity of symptoms in patients with jumper's knee (patellar tendinosis). Victorian Institute of Sport Tendon Study Group. *J. Sci. Med. Sport* **1**, 22–8 (1998).
42. Pham, T. & Tubach, F. Patient acceptable symptomatic state (PASS). *Jt. Bone Spine* **76**, 321–323 (2009).
43. Purdam, C. R. *et al.* Discriminative ability of functional loading tests for adolescent jumper's knee. *Phys. Ther. Sport* **4**, 3–9 (2003).
44. Bojsen-Møller, J., Magnusson, S. P., Rasmussen, L. R., Kjaer, M. & Aagaard, P. Muscle performance during maximal isometric and dynamic contractions is influenced by the stiffness of the tendinous structures. *J. Appl. Physiol.* **99**, 986–94 (2005).
45. Silbernagel, K. G., Gustavsson, A., Thomeé, R. & Karlsson, J. Evaluation of lower leg function in patients with Achilles tendinopathy. *Knee Surgery, Sport. Traumatol. Arthrosc.* **14**, 1207–1217 (2006).
46. Taş, S., Onur, M. R., Yılmaz, S., Soylu, A. R. & Korkusuz, F. Shear Wave Elastography Is a Reliable and Repeatable Method for Measuring the Elastic Modulus of the Rectus Femoris Muscle and Patellar Tendon. *J. Ultrasound Med.* **36**, 565–570 (2017).
47. Regensburger, A. P. *et al.* Detection of collagens by multispectral optoacoustic tomography as an imaging biomarker for Duchenne muscular dystrophy. *Nat. Med.* **25**, 1905–1915 (2019).
48. Kregel, J., van Wilgen, C. P. & Zwerver, J. Pain assessment in patellar tendinopathy using pain pressure threshold algometry: An observational study. *Pain Med. (United States)* **14**, 1769–1775 (2013).
