# Supplementary material for: Extended Restitution Between Sessions Does Not Enhance the Benefits of 12 Weeks Exercise‐Based Treatment for Patellar Tendinopathy: A Randomized Controlled Clinical Trial (The TEREX Trial)
Source: Scand J Med Sci Sports. 2026 Mar 8;36(3):e70235. doi: 10.1111/sms.70235 (PMC12968374; doi:10.1111/sms.70235)
Supplement: Supplementary file 3 — Table S1: Baselines characteristics. Table S2: Clinical Results. Table S3: Sports participation (hr/wk). Table S4: Satisfaction and Improvement. Table S5: Functional results. Table S6: Ultrasonography findings injured leg. Figure S1: Clinical improvement in VISA‐P. Values are presented as mean ± SEM at baseline (0wk) and after intervention (12wk) for the two intervention groups. VISA‐P, Victorian Institute of Sports Assessment–Patella, SR, Short restitution group; ER, Extended restitution group. Mixed effect model was performed for all analysis with time and group as main factors. Alpha level set at p < 0.05. P‐values group (0.16), time (< 0.0001), and interaction (0.54). Figure S2: Figures showing individual participant data for VISA‐P, VISA‐P truncated, NRS SLDS, and NRS Daily activity. VISA‐P, Victorian Institute of Sports Assessment–Patella; SLDS, Single leg decline squat; Short restitution group; ER, Extended restitution group. [file SMS-36-e70235-s001.pdf]

Supplementary Material:

Results Per Protocol analysis:

Table S1. Baselines characteristics

**Variable**	**SR** (n=21)	**ER**(n=23)
Age, y	32.0 ± 8.7 (21-56)	33.4 ± 9.1 (22-54)
Male/female, n	14/7	19/4
Height, cm	180.4 ± 8.5	181.9 ± 8.2
Weight, kg	77.7 ± 13.0	87.9 ± 16.5
Body mass index (kg/m^2)	23.7 ± 2.2	26.4 ± 3.5
Symptom duration, mo	12.2 ± 6.5 (5-24)	11.8 ± 7.1 (4-24)
Sport participation, h/wk	8.1 ± 4.8 (2-20)	8.3 ± 3.6 (2-15)
Unilateral/bilateral injury, n	13 / 8	10 / 13

Values are expressed as mean ± SD (range) unless otherwise noted. SR, Short restitution group; ER, Extended restitution group

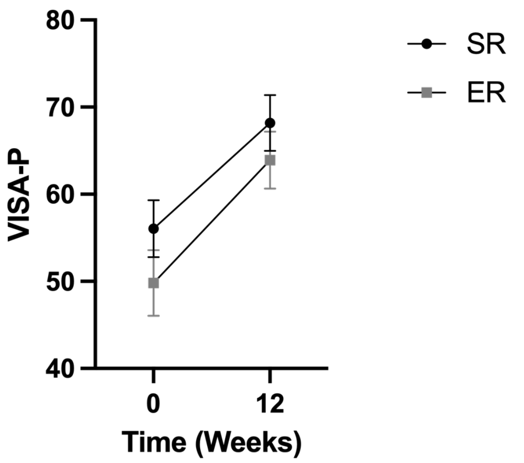

Figure S1. Clinical improvement in VISA-P. Values are presented as mean±SEM at baseline (0wk) and after intervention (12wk) for the two intervention groups. VISA-P, Victorian Institute of Sports Assessment–Patella, SR, Short restitution group; ER, Extended restitution group. Mixed effect model was performed for all analysis with time and group as main factors. Alpha level set at P <0.05. P-values group (0.16), time (<0.0001), and interaction (0.54).

TABLE S2 : Clinical Results

	**SR** (n=21)	**ER** (n=23)	*P* Value		
			Group	Time	Group x Time
**VISA-P**, Point					
0 weeks	56.0 ± 3.3 (49.2 to 62.9)	49.8 ± 3.8 (42.0 to 57.7)	0.24	<0.0001	0.63
12 weeks	68.2 ± 3.2 (61.5 to 74.9)	63.9 ± 3.3 (57.2 to 70.7)			
**VISA-P truncated**, Point					
0 weeks	31.4 ± 2.3 (26.8 to 36.1)	28.8 ± 2.2 (24.3 to 33.3)	0.22	<0.0001	0.72
12 weeks	42.5 ± 1.3 (39.7 to 45.2)	39.0 ± 1.6 (39.0 to 42.4)			
**SLDS,** NRS					
0 weeks	5.7 ± 0.7 (4.3 to 7.0)	6.4 ± 0.5 (5.3 to 7.4)	0.20	<0.0001	0.30
12 weeks	2.8 ± 0.6 (1.5 to 4.0)	4.1 ± 0.6 (2.9 to 5.3)			
**Pain,** NRS					
Physical activity					
0 weeks	5.8 ± 2.3 (4.7 to 6.8)	6.3 ± 2.4 (5.3 to 7.4)	0.29	<0.0001	0.93
12 weeks	2.7 ± 1.1 (2.2 to 3.2)	3.3 ± 1.9 (2.4 to 4.1)			
Daily activity					
0 weeks	3.7 ± 0.4 (2.8. to 4.6)	4.5 ± 0.5 (3.4 to 5.6)	0.10	<0.0001	0.64
12 weeks	1.5 ± 0.3 (0.9 to 2.0)	2.6 ± 0.5 (1.5 to 3.4)			
Rest					
0 weeks	2.7 ± 0.5 (1.6 to 3.8)	3.3 ± 0.5 (2.3 to 4.5)	0.17	<0.0001	0.79
12 weeks	1.0 ± 0.2 (0.5 to 1.4)	1.8 ± 0.4 (1.0 to 2.6)			
CMJ (bilat)					
0 weeks	2.6 ± 0.5 (1.6, 3.6)	2.7 ± 0.6 (1.6, 3.9)	0.32	<0.0001	0.38
12 weeks	0.7 ± 0.3 (0.1, 1.2)	1.5 ± 0.3 (0.8, 2.2)			
CMJ (test Leg)					
0 weeks	3.0 ± 0.4 (2.1, 3.9)	4.2 ± 0.6 (2.9, 5.4)	0.04*	<0.0001	0.82
12 weeks	0.8 ± 0.2 (0.3, 1.2)	1.8 ± 0.4 (1.0, 2.6)			
Muscle strength tets (test leg)					
0 weeks	3.9± 0.6 (2.7, 5.1)	4.4 ± 0.6 (3.1, 5.6)	0.17	<0.0001	0.20
12 weeks	1.5 ± 0.3 (0.7, 2.2)	2.8 ± 0.4 (1.9, 3.7)			

Values are presented as least mean±SEM (95% CI). Mixed effect model was performed for all analysis with time and group as main factors. Alpha level set at P <0.05. SR, Short restitution group; ER, Extended restitution group; VISA-P, Victorian Institute of Sports Assessment- Patella; SLDS, single-leg decline squat NRS, numeric rating scale, CMJ, countermovement jump.

TABLE S3: Sports participation (hr/wk)

	SR (n=21)	ER (n=23)	P- Value		
			Group	Time	Group x Time
Before injury	7.8 ± 0.8 (6.2, 9.5)	8.5 ± 1.0 (6.6, 10.4)	0.67	<0.0001	0.89
0 weeks	5.6 ± 0.9 (7.6, 3.6)	6.0 ± 0.8 (4.4, 7.6)			
12 weeks	4.1 ± 0.8 (2.5, 5.7)	4.2 ± 0.6 (5.4, 3.1)			
Δ before injury to 0 wk	2.2 ± 0.9 (0.1, 4.3)	2.5 ± 0.9 (0.6, 4.5)			
Δ before injury to 12 wk	3.7 ± 0.9 (1.6, 5.8)	4.3 ± 0.9 (2.3, 6.3)			

Values are presented as least mean±SEM (95% CI). Mixed effect model was performed for all analysis with time and group as main factors. Alpha level set at P <0.05. SR, Short restitution group; ER, Extended restitution group; Δ, change in time interval.

TABLE S4 : Satisfaction and Improvement

	SR (n=21)	ER (n=23)	P- Value (group)
**Satisfaction with function**			
Daily activity			
0 weeks	8 (38)	8 (35)	>0.9999
12 weeks	14(67)	14 (61)	0.76
P-Value (time)	0.12	0.14	
Sport and physical activity			
0 weeks	1 (5)	0 (0)	0.48
12 weeks	5 (24)	4 (17)	0.72
P-Value (time)	0.18	0.05*	
Treatment satisfaction			
12 weeks	21 (100)	23 (100)	>0.9999
**GROC**			
12 weeks	21 (100)	19(83)	0.11

Values are presented as least n (%). Chi-square (and Fisher's exact) test was performed for all analysis based on a contingency table. Alpha level set at P <0.05. SR, Short restitution group; ER, Extended restitution group; GROC, Global rating of change.

TABLE S5 : Functional results

	**SR** (n=21)	**ER** (n=23)	P- Value		
			Group	Time	Group x Time
**Muscle strength**, n*m					
0 weeks	165.1 ± 10.3 (143.7 to 186.5)	182.3 ± 11.1 (160.2 to 206.3)	0.33	<0.0001	0.59
12 weeks	202.8 ± 11.7 (178.4 to 227.2)	215.5 ± 12.9 (188.7 to242.3)			
**CMJ height bilat, cm**					
0 weeks	27.6 ± 1.4 (24.6 to 30.5)	30.7 ± 1.7 (27.1 to 34.2)	3.61	0.18	0.05
12 weeks	27.3 ± 1.2 (24.7 to 29.9)	29.7 ± 1.7 (26.1 to 33.3)			
**CMJ height injured site, cm**					
0 weeks	10.8 ± 0.9 (9.1 to 12.6)	16.7 ± 4.6 (26.1 to 14.3)	1.91	0.66	1.20
12 weeks	11.5 ± 0.8 (9.7 to 13.3)	12.2 ± 1.0 (14.2 to 10.1)			

Values are presented as least mean±SEM (95% CI). Mixed effect model was performed for all analysis with time and group as main factors. Alpha level set at P <0.05. SR, Short restitution group; ER, Extended restitution group; CMJ, Counter movement jump.

TABLE S6 : Ultrasonography findings injured leg

	**SR** (n=21)	**ER** (n=23)	P- Value		
			Group	Time	Group x Time
**Power Doppler area,** mm^2					
0 weeks	33.2 ± 7.5 (17.4, 48.9)	37.4± 6.9 (23.2, 51.7)	0.55	0.15	0.59
12 weeks	27.6 ± 7.6 (11.9, 43.5)	34.9 ± 6.5 (21.5, 48.3)			
**Tendon Thickness,** mm					
0 weeks	6.9 ± 0.4 (6.0, 7.8)	7.6 ± 0.3 (6.9, 8.3)	0.34	0.41	0.19
12 weeks	7.0 ± 0.5 (5.8, 8.1)	7.3 ± 0.3 (6.7, 8.0)			

Values are presented as least mean±SEM (95% CI). Mixed effect model was performed for all analysis with time and group as main factors. Alpha level set at P <0.05. SR, Short restitution group; ER, Extended restitution group.

Figures showing individual participant data

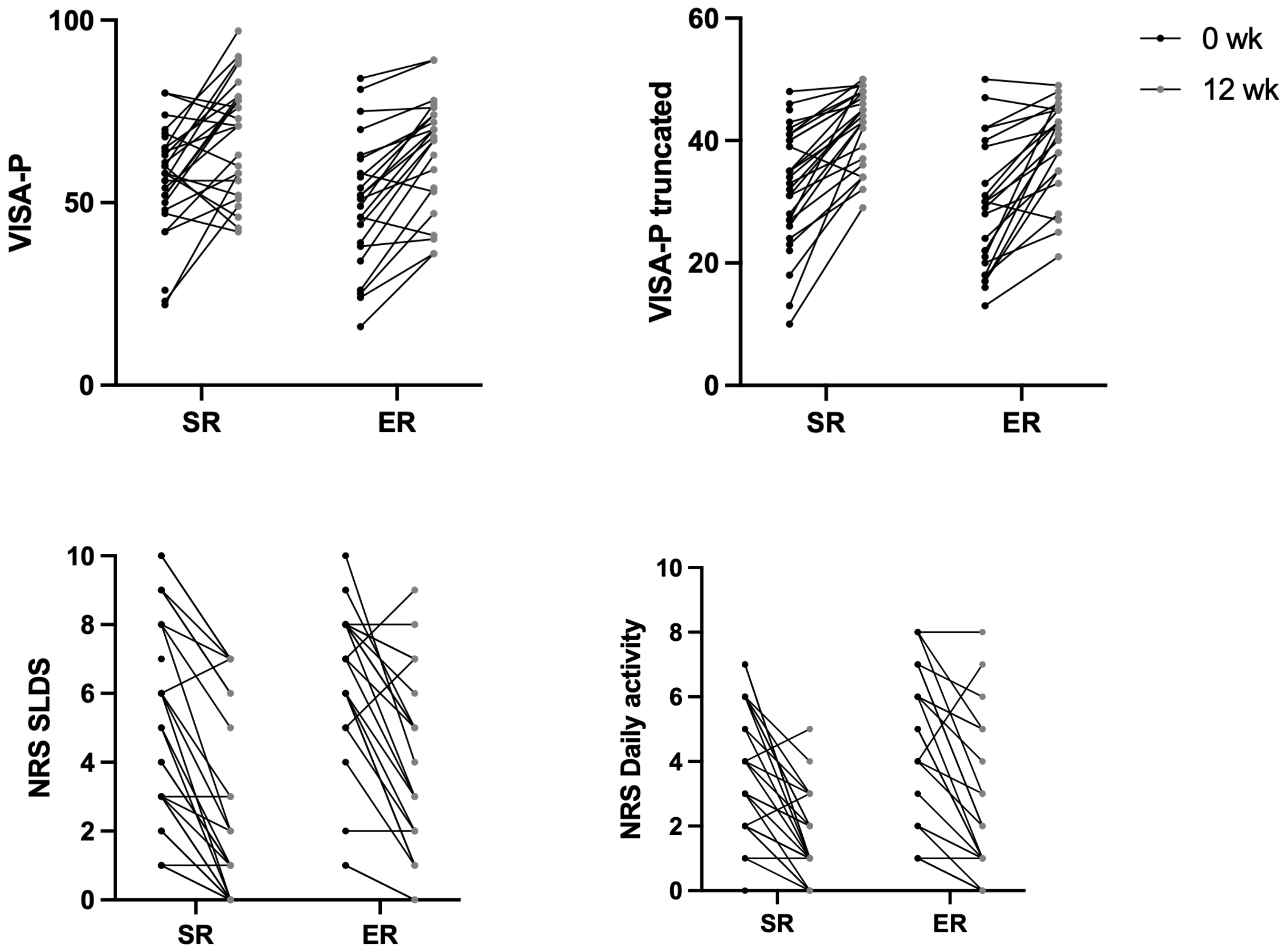

Figure S2. Figures showing individual participant data for VISA-P, VISA-P truncated, NRS SLDS, and NRS Daily activity. VISA-P, Victorian Institute of Sports Assessment–Patella; SLDS, Single leg decline squat; Short restitution group; ER, Extended restitution group.
